# Dopamine-independent effect of rewards on choices through hidden-state inference

**DOI:** 10.1038/s41593-023-01542-x

**Published:** 2024-01-12

**Authors:** Marta Blanco-Pozo, Thomas Akam, Mark E. Walton

**Affiliations:** 1 Department of Experimental Psychology, Oxford University, Oxford, UK; 2 Wellcome Centre for Integrative Neuroimaging, Oxford University, Oxford, UK

**Keywords:** Decision, Motivation, Motivation, Reward

## Abstract

Dopamine is implicated in adaptive behavior through reward prediction error (RPE) signals that update value estimates. There is also accumulating evidence that animals in structured environments can use inference processes to facilitate behavioral flexibility. However, it is unclear how these two accounts of reward-guided decision-making should be integrated. Using a two-step task for mice, we show that dopamine reports RPEs using value information inferred from task structure knowledge, alongside information about reward rate and movement. Nonetheless, although rewards strongly influenced choices and dopamine activity, neither activating nor inhibiting dopamine neurons at trial outcome affected future choice. These data were recapitulated by a neural network model where cortex learned to track hidden task states by predicting observations, while basal ganglia learned values and actions via RPEs. This shows that the influence of rewards on choices can stem from dopamine-independent information they convey about the world’s state, not the dopaminergic RPEs they produce.

## Abstract (web summary)

Reward can both update values and convey information about the state of the world. Dopamine recordings and manipulations in highly trained mice making decisions, alongside modeling, show that dopamine supports the former but not the latter process.

## Main

Adaptive behavior requires learning which actions lead to desired outcomes and updating this knowledge when the world changes. Reinforcement learning (RL) has provided an influential account of how this works in the brain, with RPEs updating estimates of the values of states and/or actions, in turn driving choices. In support of this framework, dopamine activity resembles RPEs in many behaviors^1–4^, and causal manipulations can reinforce or suppress behaviors consistent with dopamine acting functionally as an RPE^5–8^.

However, value learning is not the only way we adapt to changes in the environment. For example, we behave differently on weekdays and weekends, but this is clearly not because we relearn the value of going to work versus spending time with family each Saturday morning. Rather, although the world looks the same when we wake up, we understand that the state of the world is in fact different, and this calls for different behavior. Formally, the decision problem we face is partially observable—our current sensory observations only partially constrain the true state of the world. In such environments, it is typically possible to estimate the current state better using the history of observations than using just the current sensory input^9,10^.

It is increasingly clear that this ability to infer hidden (that is, not directly observable) states of the world plays an important role even in simple laboratory reward-guided decision-making^10–18^. For example, in probabilistic reversal learning tasks where reward probabilities of two options are anticorrelated, both behavior and brain activity indicate that subjects understand this statistical relationship^10,12,13,19^. This is not predicted by standard RL models in which RPEs update the value of preceding actions, but is predicted by models which assume subjects understand there is a hidden state that controls both reward probabilities. Intriguingly, brain recordings have shown that not only prefrontal cortex (PFC) but also the dopamine system can reflect knowledge of such hidden states^13,19–23^.

Integrating these two accounts of behavioral flexibility raises several pressing questions. If state inference, not RL, mediates flexible reward-guided behavior, why does dopamine look and act like an RPE? Conversely, if value updates driven by dopaminergic RPEs are responsible, how does this generate the signatures of hidden-state inference seen in the data?

To address these questions, we measured and manipulated dopamine activity in highly trained mice performing a two-step decision task. The task had two important features. First, reward probabilities were anticorrelated and reversed periodically, constituting a hidden state that could be inferred by observing where rewards were obtained. Second, inference and RL-based strategies could be differentiated by measuring how prior rewards affected dopamine activity. Behavior and dopamine signaling were consistent with mice tracking a single hidden state of the reward probabilities. However, while dopamine signals closely resembled RPEs, neither activating nor inhibiting dopamine neurons at trial outcome had any effect on subsequent choice. We show that these apparently paradoxical data can be reproduced by a neural network model in which cortex infers hidden states by predicting observations and basal ganglia uses RL mediated by dopaminergic RPEs to learn appropriate actions.

## Results

### Mice behavior respects task causal structure

**Fig. 1:**
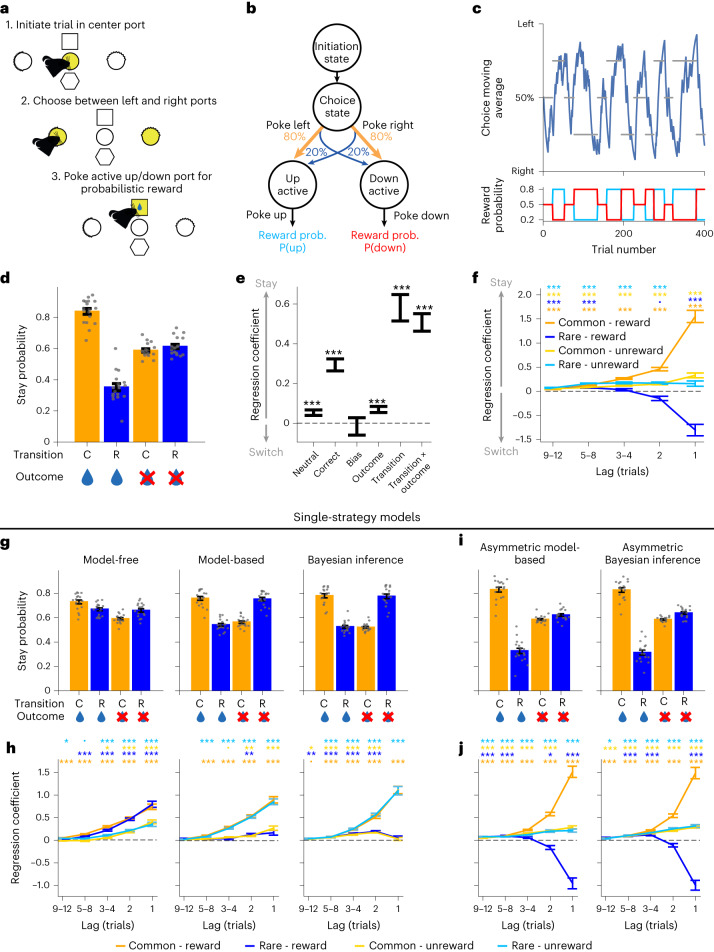
Two-step task behavior. **a**, Diagram of trial events. Each trial started with a central port lighting up, which mice poked to initiate the trial. This triggered either a choice state, where both left and right ports lit up (75% of trials), or a forced choice where either the left or the right port lit up (25% of trials). Poking an illuminated side port triggered a transition to one of two possible second-step states, signaled by a 1-s auditory cue, in which either the top or the bottom port was illuminated. Poking the active reward port caused a 500-ms auditory cue indicating the trial outcome (reward or not), with reward delivered at cue offset. **b**, Diagram of the task state space, reward and transition probabilities. **c**, Example behavioral session. Top, moving average of choices (blue trace) with reward probability blocks shown by gray bars, indicating by their vertical position whether the reward probability was higher for the state commonly reached for the left or right choice or a neutral block. Bottom, reward probability at the up (blue) and down (red) ports. **d**, Probability of a choice being repeated as a function of the subsequent state transition (common or rare) and trial outcome (rewarded or unrewarded). Dots show individual subjects; error bars indicate the cross-subject mean ± s.e.m. **e**, Mixed-effects logistic regression predicting repeating choice as a function of the correctness of the choice (high reward probability; split as correct, incorrect or neutral), choice bias (left or right), the trial outcome (rewarded or not), state transition (common or rare) and the transition–outcome interaction. Error bars indicate the mixed-effects model estimate ± s.d.; statistical significance was assessed using a likelihood-ratio test with type 3 sums of squares. **f**, Lagged logistic predicting choice as a function of the history of different trial types defined by the transition and outcome. This strong asymmetry between the effect of reward and reward omission is surprising given that both outcomes are equally informative about future reward, but might reflect differences between task statistics and the demands of foraging in natural environments. Error bars indicate the cross-subject mean ± s.e.m. **a**–**f**, *n* = 18 mice, with a total 181,323 trials in 520 sessions; statistical significance was assessed using a two-sided *t*-test comparing the cross-subject distribution against zero, Bonferroni corrected. **g**–**j**, Single-strategy models: stay probability (as in **d**), error bars indicate the cross-subject mean ± s.e.m. (**g** and **i**); and lagged regression (as in **f**), error bars indicate the cross-subject mean ± s.e.m.; statistical significance was assessed using a two-sided *t*-test comparing the cross-subject distribution against zero, Bonferroni corrected (**h** and **j**), of simulated behavior from different RL models fitted to mouse behavior. **g**–**j**, *N* = 18 simulated subjects, with numbers of trials and sessions per subject matched to the corresponding mouse. *P < 0.05, **P < 0.01, ***P < 0.001. For exact *P* values, see Supplementary Table 5.

We trained dopamine transporter (DAT)-Cre mice (*n* = 18) on a sequential decision task, which required them to choose between two options—left and right—to gain access to one of two reward ports—up or down (Fig. 1a). Each first-step choice led commonly (80% of trials) to one second-step state and rarely (20% of trials) to the other (Fig. 1b). Reward probabilities in each port changed in blocks between 0.8/0.2, 0.5/0.5 and 0.2/0.8 on the up/down ports, respectively (Fig. 1c), and were therefore anticorrelated at the up and down ports, while transition probabilities remained fixed. This facilitates hidden-state inference strategies (also referred to as ‘latent-state’ inference^24^) because the state of the reward probabilities—whether up or down is rewarded with higher probability—fully determines which first-step action is best^24,25^.

Subjects tracked which option was currently best, performing 348.70 ± 93.90 trials and completing 8.32 ± 3.24 blocks per session (mean ± s.d. across subjects; Fig. 1c). Reward following a common transition promoted repeating the same first-step choice on the next trial, while reward following a rare transition promoted switching to the other first-step choice (Fig. 1d,e; mixed-effects logistic regression—transition × outcome: *β* = 0.507, s.e. = 0.044, *z* = 11.468, *P* < 0.001), and these effects persisted over multiple trials (Fig. 1f). This pattern is adaptive because it corresponds to rewards promoting choice of first-step actions that commonly lead to the second-step states where they were obtained. However, the probability of repeating the same choice following a non-rewarded outcome was similar irrespective of whether a common or rare transition occurred (Fig. 1d,f).

**Extended Data Fig. 1:**
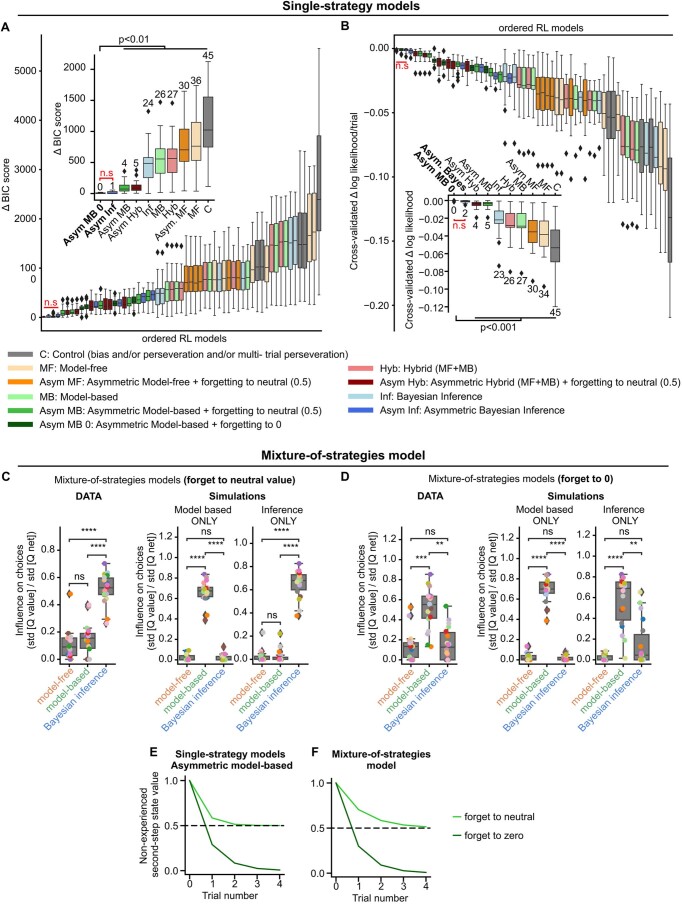
Model comparison and mixture-of-strategies model. **A-B)** Model-comparison for single-strategy models; statistical significance, two-sided Wilcoxon signed-rank test (with normal approximation due to ties) with Bonferroni correction. **a)** BIC score model-comparison of all the models tested (see Supplementary Table 1 for details of each model). Colours indicate the model’s strategy. Each strategy was tested both with and without choice bias and multi-trial perseveration. Additionally, models were tested with both symmetric and asymmetric learning from reward and omission outcomes (see Methods). Model-free and model-based RL strategies were tested with and without forgetting about the values of not-visited states and not-chosen actions. Two variants of forgetting were tested: one implemented forgetting as value decay towards a neutral value of 0.5, the other as value decay towards 0. Insets show the best-fitting model of each model class. Fitted parameter values for the best model in each class are presented in Supplementary Table 2. **b)** Cross-validated log likelihood of the same models as in **A**. Inset, as in **A**, best fitting model of each category. ***Both model comparison approaches indicate that asymmetric inference and asymmetric model-based RL with forgetting to 0 strategies explain the data equally well, and better than other strategies considered***. **c-f)** Mixture-of-strategies model: choices were determined by a weighted combination of the asymmetric versions of model-free RL, model-based RL, and Bayesian inference. We tested two different mixture-of-strategies models differentiated by how forgetting was implemented for the RL components: In **C)** forgetting was implemented as value to decay towards a neutral value of 0.5, while in **D)** forgetting was implemented as value decay towards 0. **C)** Mixture-of-strategies model with forgetting towards neutral value. Left panel: influence of each model component on choices, evaluated using the fit of the model to each subject’s data. The influence on choices was quantified as the standard deviation across trials of the difference between the two first-step action values due to a given component, divided by the total standard deviation of the value difference due to all components. Each coloured dot represents a single mouse. Centre and Right panels: influence of each model component for fits of the mixture-of-strategies model to behaviour simulated from either a model-based (centre panel) or Bayesian inference (right panel) single-strategy model with parameters fit to each subject’s choices. The fit correctly assigned high weight to the strategy that generated the simulated data and low weight to the other strategies. **D)** As in **C** but with forgetting towards 0. When forgetting was implemented as decay towards 0, the mixture-of-strategies model fit to simulated data did not correctly identify which strategy generated simulated data. ***Therefore, consistent with the model comparison, we cannot arbitrate between asymmetric inference and asymmetric model-based RL with forgetting to 0 strategies using the mixture-of-strategies model****.* Statistical significance, two-sided paired t-test, Bonferroni corrected. **E,F)** Value decay of the non-experienced second-step state over consecutive trials when forgetting decayed towards neutral (light green) or zero (dark green) in **E)** the asymmetric model-based model and **F)** in the mixture-of-strategies model. Boxplots show the distribution of the data across subjects. Grey box represents the interquartile range, with horizontal lines representing first quartile, median and third quartile, from bottom to top. Whiskers represent minimum and maximum values. Rhomboids mark outliers. N=18 simulated subjects, numbers of trials and sessions per subject matched to corresponding mouse. n.s. non-significant, *P<0.05, **P<0.01, ***P<0.001. For exact p-values, see Supplementary Table 6.

To assess what strategy the animals used, we fitted a set of models to their choices and simulated data from the fitted models (Fig. 1g–j). Neither model-free nor model-based RL, commonly used to model two-step task behavior^26^, resembled subject’s choices (Fig. 1g,h). We also considered a strategy that used Bayesian inference to track the hidden state of the reward probabilities, combined with a fixed habit-like mapping from this state estimate to the corresponding high-value first-step action^24^, but again this did not resemble the experimental data. These models failed because both predict a symmetric influence of reward and omission on choices, contrary to our experimental data; therefore, we modified each model to incorporate this asymmetry. For model-based RL, this was done using different learning rates for positive and negative RPEs. We also incorporated forgetting about the value of states that were not visited, as this was supported by model comparison (Extended Data Fig. 1). This approach was not possible for the inference model, as Bayesian updates do not have a learning rate parameter that can be different for reward and omission. We therefore implemented the asymmetry by modifying the observations received by the model, so that it treated reward obtained in the two second-step states as different observations, but treated reward omission as the same observation irrespective of the state where it occurred. Simulated on the task, both asymmetric models generated a pattern of stay probabilities that closely matched subject’s data (Fig. 1i,j).

We adopted two different approaches to try and differentiate between these strategies: (i) likelihood-based model comparison (Supplementary Tables 1 and 2), and (ii) fitting a mixture-of-strategies model incorporating both components to assess which explained most variance in subjects’ choices (Supplementary Table 3). Both analyses gave a consistent picture that it was not possible to arbitrate between the strategies using behavior alone (Extended Data Fig. 1). Critically, however, the two strategies make different predictions for how rewards update the estimated value of each second-step state (discussed below), and hence for dopaminergic RPE signaling. We therefore looked for evidence of inference-based value updates in dopamine activity.

### Inferred values drive dopamine signals

**Fig. 2:**
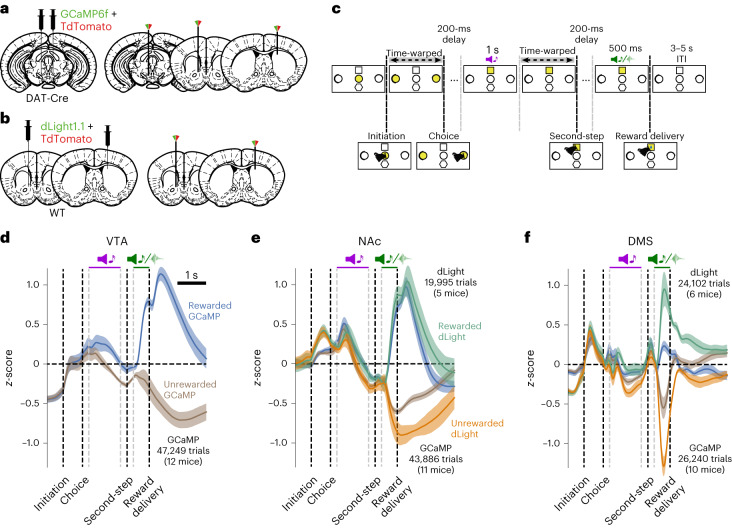
Dopamine activity and release are modulated across the trial. **a**,**b**, Injection and implant schematic. **a**, For calcium photometry, DAT-Cre mice were injected bilaterally with GCaMP6f and TdTomato in VTA, and optical fibers were implanted in the VTA and NAc in one hemisphere, and the DMS in the other hemisphere. **b**, For dLight photometry, wild-type mice were injected with dLight1.1 and TdTomato in NAc and DMS in different hemispheres, and optical fibers implanted at the same sites. **c**, Trial timeline. Trials were aligned by warping the intervals between the trial initiation and choice, and between the second-step port lighting up and being poked. **d**–**f**, Mean *z*-scored calcium signal and dopamine receptor binding on rewarded and unrewarded trials with shaded areas indicating the cross-subject s.e.m. in VTA GCaMP (*n* = 12 mice, 121 sessions, 47,249 trials; **d**), NAc GCaMP (*n* = 11 mice, 108 sessions, 43,886 trials; **e**) and dLight (*n* = 5 mice, 64 sessions, 19,995 trials; **e**) and DMS GCaMP (*n* = 10 mice, 74 sessions, 26,240 trials; **f**) and dLight (*n* = 6 mice, 91 sessions, 24,102 trials; **f**).

**Extended Data Fig. 2:**
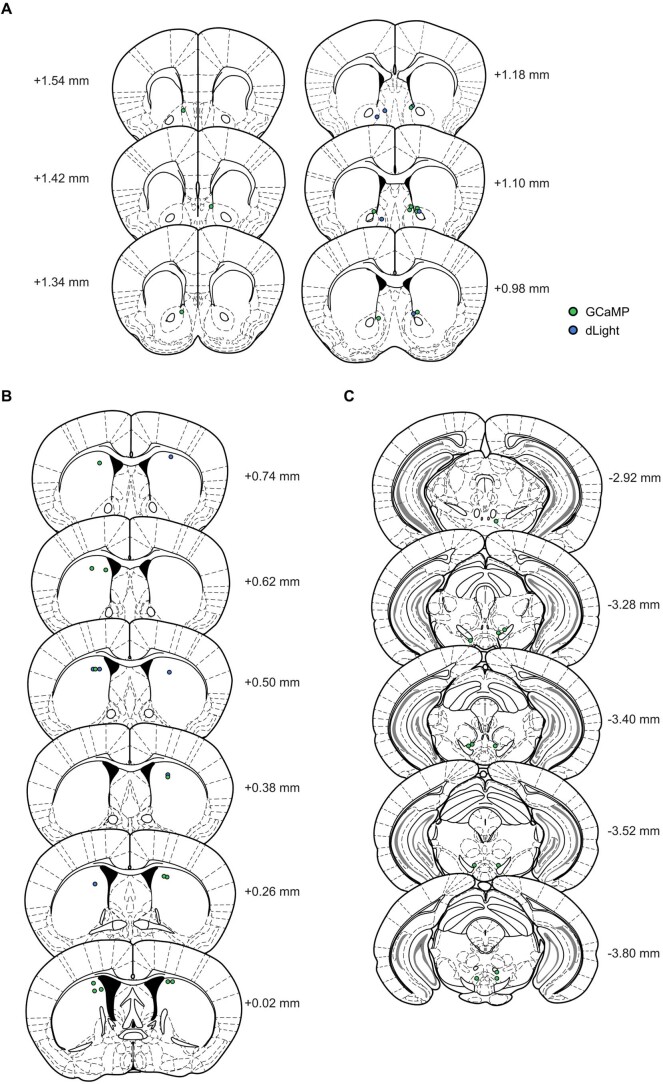
Fibre placement for photometry experiments. **a)** NAc, **b)** DMS, and **c)** VTA. Green, GCaMP6f; blue, dLight1.1 animals.

**Extended Data Fig. 3:**
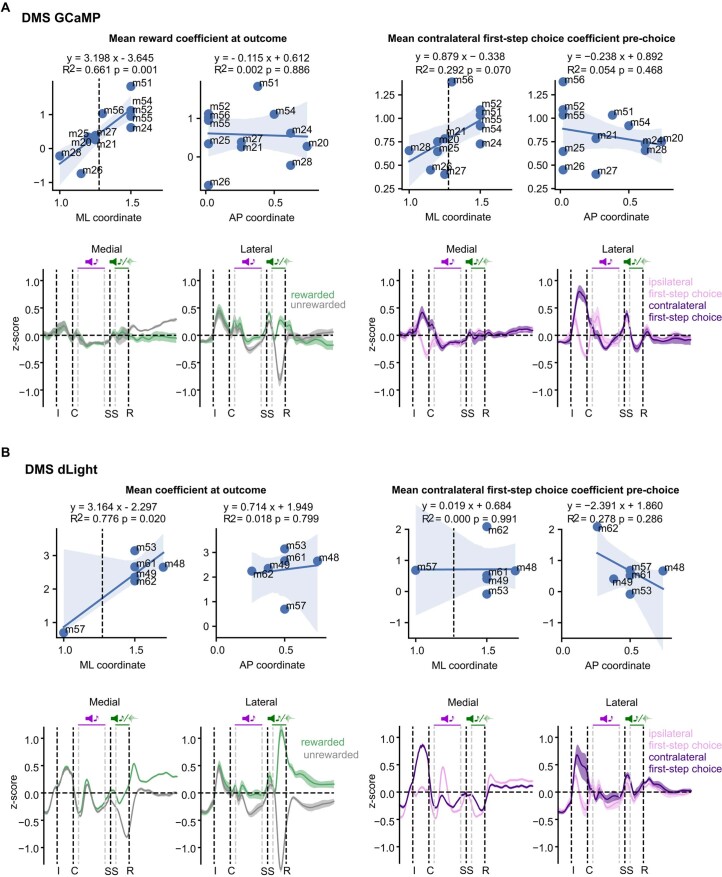
Medio-lateral gradient in reward modulation in DMS. **a)** Dopamine activity measured through GCaMP. **b)** Dopamine receptor binding measured through dLight. *Left*, mean reward coefficient (from the linear regression model) at the time of outcome cue; *Right*, mean contralateral choice coefficient pre-choice. *Top*, correlation between mean coefficient and medio-lateral (ML) or antero-posterior (AP) location of the optic fibre. Two-sided Wald test against 0. *Bottom*, mean z-scored activity split by outcome or lateralised choice for animals with medial or lateral placement in DMS. Dashed line in the correlation plots shows how animals were divided into two groups (medial and lateral).

We used fiber photometry to record calcium signals from GCaMP6f-expressing dopamine neuron cell bodies in the ventral tegmental area (VTA) and axons in the nucleus accumbens (NAc) and the dorsomedial striatum (DMS; Fig. 2a), and dopamine release using dLight1.1 expressed pan-neuronally in the NAc and DMS (Fig. 2b; see Extended Data Fig. 2 for placements). Dopamine activity fluctuated dynamically across the trial, as mice made their initial choice and received information about the second-step state reached and the trial outcome (Fig. 2d–f). Reward responses were prominent in all signals, although relatively weaker in DMS calcium. However, average DMS calcium activity masked a strong mediolateral gradient in reward response, with larger responses more laterally in the DMS (Extended Data Fig. 3). For the following analyses, we excluded the DMS site in two animals where the fiber was most medial, and we observed a negative reward response (Extended Data Fig. 3).

**Fig. 3:**
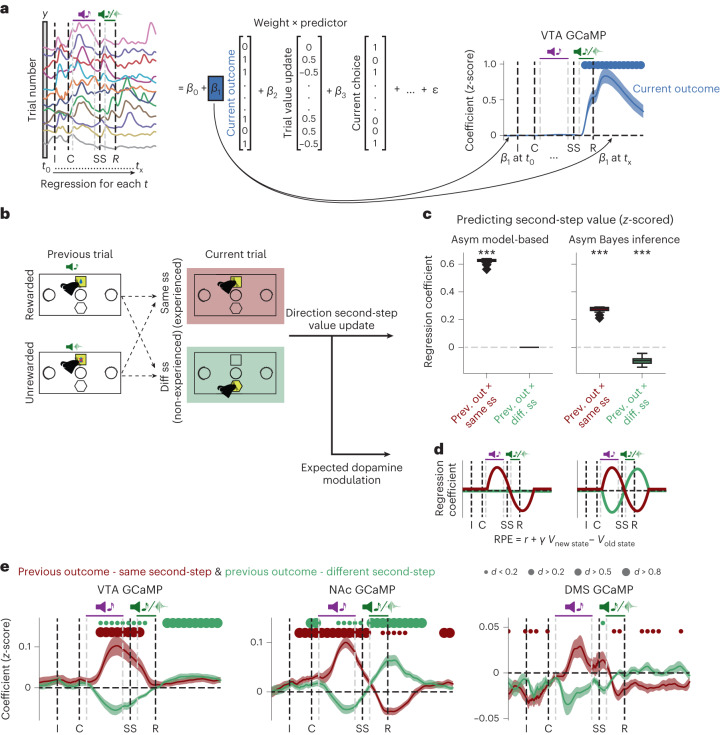
Inferred value information drives dopamine activity. **a**, Left, schematic of the linear regression model predicting dopamine activity for each timepoint in the trial. Right, coefficients in the linear regression for the current trial outcome predictor in VTA GCaMP signal, showing the cross-subject mean and s.e. Dots indicate effect size at timepoints where coefficients are statistically significant, assessed by a two-sided *t*-test comparing the cross-subject distribution against zero, Benjamini–Hochberg corrected for comparison of multiple timepoints. **b**, Schematic of the two regressors used to test the influence of previous trial outcome on the value of the same (dark red) or different (green) second-step state value reached on the current trial. **c**, Linear regression predicting second-step state value as a function of the previous trial outcome, and whether the current trial second-step state was the same or different from the previous trial, for the model-based (asym model-based) and Bayesian inference (asym Bayes inference) strategies. The regression also included the other predictors used to explain the photometry signal in **a**. Box plots show the distribution of cross-subjects regression estimates; the box represents the interquartile range, with horizontal lines representing the first quartile, median and third quartile, from bottom to top. Whiskers represent minimum and maximum values. Rhomboids mark outliers. Statistical significance was assessed using a two-sided *t*-test against zero, Bonferroni corrected, ***P = 3.88 × 10^−27^ (asym model-based); *P* = 4.74 × 10^−20^, *P* = 1.02 × 10^−10^ (asym Bayes inference, same second-step or different second-step respectively). **d**, Predicted dopamine modulation in the linear regression in **a**, based on the direction of the second-step value update from **c** under the assumption that dopamine modulation is consistent with the canonical RPE framework (where the value of the new state—that is, second-state value at second-step cue—has a positive influence on dopamine activity, while the value of the previous state—that is, second-state value at outcome time—has a negative influence on dopamine activity). **e**, Coefficients in the linear regression predicting dopamine activity for the predictors in **b**, showing the influence of previous trial outcome when the second-step state was the same (dark red) or different (green) from the previous trial in the VTA, NAc and DMS (See Extended Data Figs. 4 and 5 for dopamine concentrations; dLight). The shaded area as in **a** indicates the cross-subject mean and s.e.m. Dots indicate effect size of the statistically significant timepoints, Benjamini–Hochberg corrected. Photometry group numbers are the same as in Fig. 2.

A key feature of the state inference strategy is that it assumes that a single hidden variable controls both reward probabilities. Therefore, reward obtained in one second-step state not only increases the value of that state but also decreases the value of the other second-step state, unlike in standard model-based RL where the state values are learned independently (Fig. 3c). We can therefore leverage our photometry data to discriminate between these strategies by examining how the previous trial’s outcome influences dopamine activity and release when the second-step state reached on the current trial is revealed. Specifically, we can ask whether a reward obtained in one second-step state (for example, up-active), decreases the dopamine response to the other second-step step state (down-active*)* if it is reached on the next trial.

**Extended Data Fig. 4:**
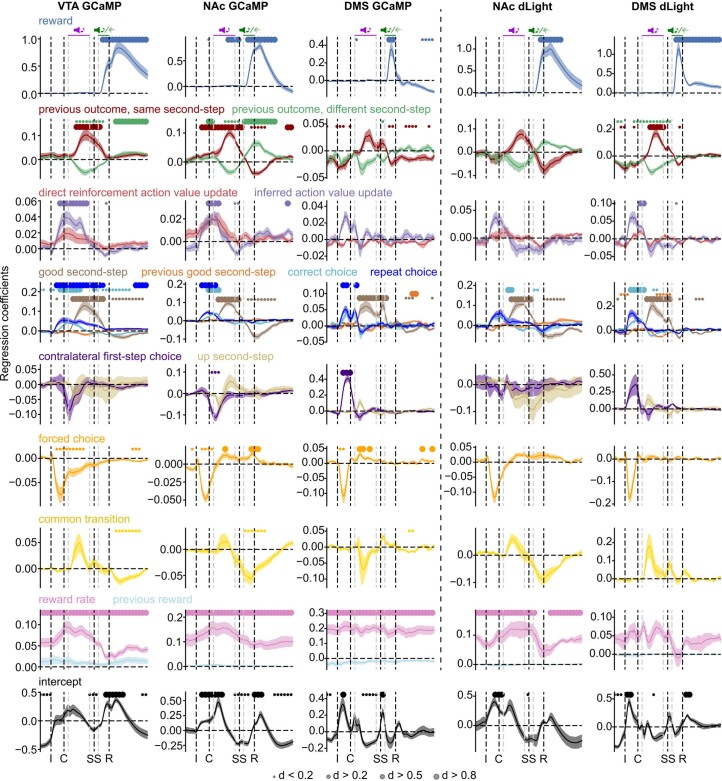
Regression coefficients from all the predictors used in the behavioural logistic regression model predicting VTA, NAc and DMS dopamine activity (GCaMP recordings) and NAc and DMS dopamine receptor binding (dLight recordings). Traces reflect mean regression coefficients across subjects; shaded area indicates cross-subject standard error. Dots indicate effect size of the statistically significant timepoints, two-sided t-test comparing the cross-subject distribution against 0, after Benjamini-Hochberg multiple comparison correction.

We aligned activity across trials and used linear regression to model dopamine fluctuations across trials and timepoints. We ran separate regression analyses for each timepoint, using predictors that varied from trial to trial but using the same value for all timepoints in each trial. The time courses of predictor loadings across the trial, therefore, reflect when, and with what sign, each predictor explained variance in the activity (Fig. 3a). The key predictors for differentiating between strategies included one coding for the previous trial’s outcome on trials where the second-step state is the same as on the previous trial, and another coding for the previous trial’s outcome when the second step reached on the current trial was different (Fig. 3b). We also included regressors modeling the current trial outcome and other possible sources of variance (Methods and Extended Data Fig. 4). We focus on the GCaMP data in the main figures, but results from dLight were closely comparable, except where noted in the text (Extended Data Fig. 4).

When the second-step state was the same as on the previous trial, the previous trial’s outcome positively influenced dopamine when the second-step state was revealed, consistent with both model-based RL and inference (Fig. 3d,e and Extended Data Fig. 4). However, when the second-step state was different to the previous trial, the previous outcome negatively influenced dopamine when the second-step state was revealed. This is at odds with the predictions of standard model-based RL but, crucially, is consistent with inference (Fig. 3d,e and Extended Data Fig. 4). In NAc, loading on these regressors reversed sign at outcome time. This biphasic response is exactly as predicted for an RPE; RPEs are computed from value differences between successive time steps, so if dopamine reports RPE, the value of the second-step state reached on the trial will drive a positive response when the state is revealed, followed by a negative response at the time of trial outcome^27,28^. Unexpectedly, this reversal was not observed in either the VTA or the DMS.

**Extended Data Fig. 5:**
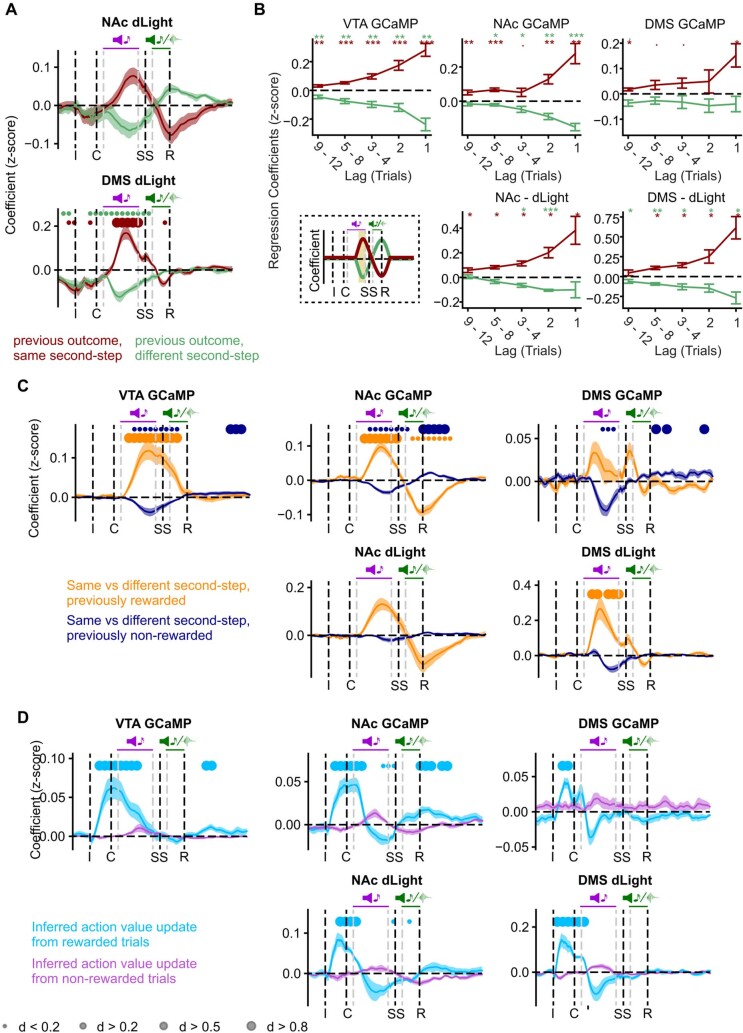
Value update regressors. **a)** Coefficients in the linear regression predicting dopamine activity showing the influence of previous trial outcome when the second-step was the *same* (dark red) or *different* (green) from the previous trial for NAc and DMS dLight signals. **b)** Linear regression predicting the dopamine response to the second-step cue as a function of the extended history of trial events over the previous 12 trials. Error bars cross subject mean ± s.e.m. Bottom left, Schematic of dopamine signal, yellow shaded area represents the predicted activity. Two-sided t-test comparing the cross-subject distribution against 0, Bonferroni corrected. For exact p-values, see Supplementary Table 6. **c)** Second-step value update regressors, whether the current second-step was the same or different from the previous trial, split by rewarded and not rewarded outcome on the previous trial. **d)** Inferred action value update split by outcome on the previous trial. Shaded area indicates cross-subject standard error. Dots indicate effect size of the statistically significant timepoints, two-sided t-test comparing the cross-subject distribution against 0, after Benjamini-Hochberg multiple comparison correction.

To ensure that this pattern of modulation by inferred value was not an artifact of confounding effects of trial history, we performed a lagged regression predicting the dopamine response to the second-step state cue. This confirmed that the dopamine response was driven by inferred state values, and that these integrated outcomes over multiple previous trials (less clearly in the DMS axonal calcium activity, but prominently in DMS dopamine release; Extended Data Fig. 5b).

To test whether the asymmetric influence of rewards and omissions on subject’s choices was also reflected in dopamine activity, we ran a modified regression analysis using separate regressors for trials following rewarded and non-rewarded outcomes. In the VTA and NAc, the differential dopamine response to reaching the same versus different second-step state was much stronger following rewarded than non-rewarded trials, consistent with rewards updating second-step state values more strongly than omissions (Extended Data Fig. 5c). Dopamine activity at choice time was also higher when subjects chose an action that commonly led to the state where reward was obtained on the previous trial (Extended Data Fig. 4, inferred action value update), consistent with subjects inferring the value of the first-step action using knowledge of the task structure. Again, these effects were primarily driven by rewarded rather than omission trials (Extended Data Fig. 5d).

Together, these findings indicate that the mice understood that a single hidden variable controlled the reward probabilities in both ports and inferred its current state by observing where rewards were obtained. Reward predictions based on this then shaped dopamine responses to both task states.

### Dissociable influence of RPE, reward rate and movement on dopamine activity

**Fig. 4:**
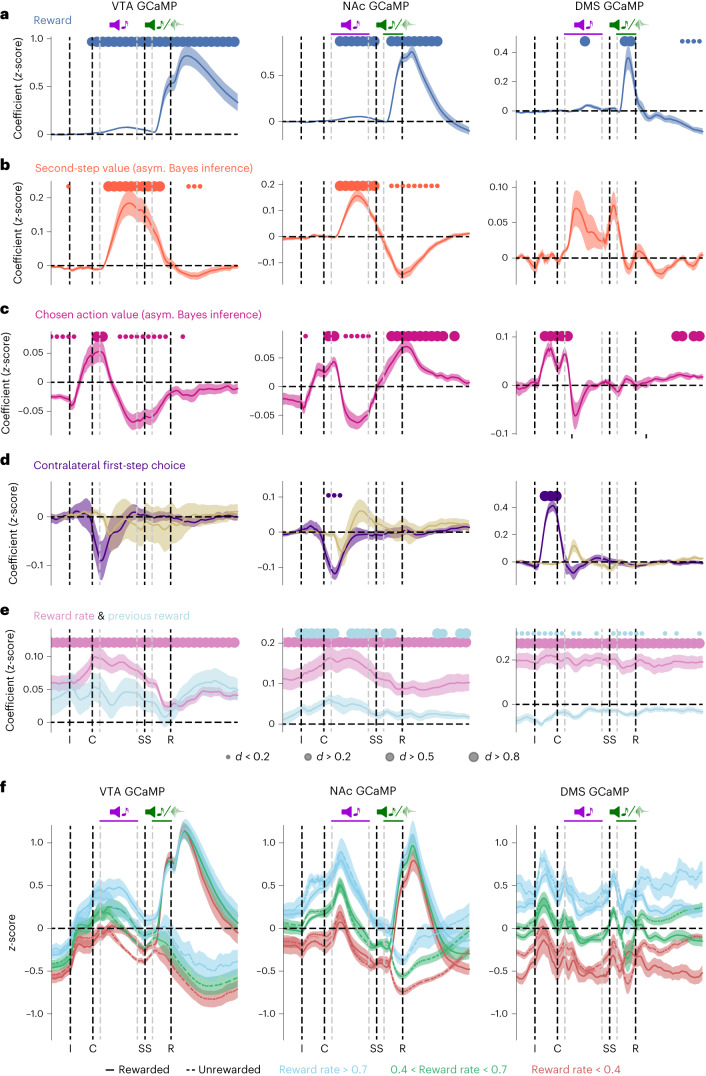
Simultaneous RPE, reward rate and lateralized movement signals in dopamine activity. **a**–**e**, Coefficients from the regression using the value estimates from the asymmetric Bayesian inference model, predicting dopamine activity for each timepoint in the trial for each region, showing the cross-subject mean and s.e.m. (shaded area). Dots indicate effect size at timepoints where coefficients are statistically significant, assessed by a two-sided *t*-test comparing the cross-subject distribution against zero, Benjamini–Hochberg corrected for comparison of multiple timepoints. **a**, Reward, current trial outcome. **b**, Value of the second step reached on the current trial derived from the asymmetric Bayesian inference model. **c**, Value of the chosen first-step action derived from the asymmetric Bayesian inference model. **d**, Contralateral first-step choice (coded according to the initial choice—left/right—relative to the hemisphere being recorded from). **e**, Recent reward rate (exponential moving average with tau = 8 trials—similar results were obtained using time constants of 5–15 trials) and previous trial outcome. **f**, Mean *z*-score activity split by rewarded/unrewarded trials and recent reward rate. Shaded area indicates the s.e.m. Blue denotes a high reward rate > 0.7 rewards/trial (exponential moving average with tau = 10 trials). Green denotes a medium reward rate (between 0.4 and 0.7). Red denotes a low reward rate (less than 0.4). Photometry group numbers are the same as in Fig. 2.

**Extended Data Fig. 6:**
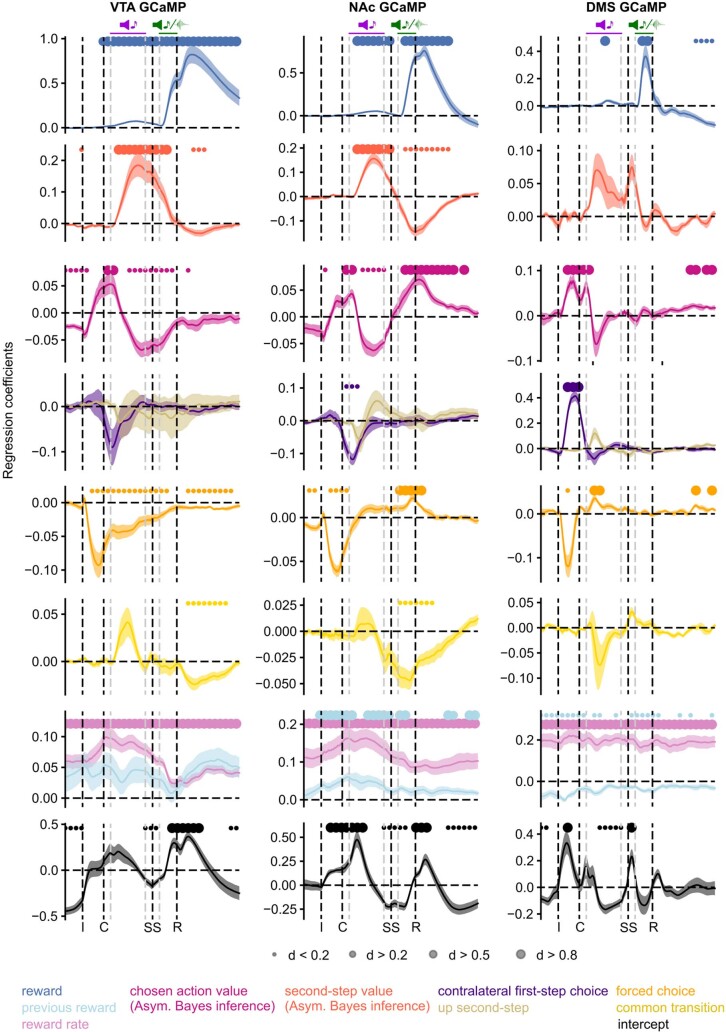
Regression model using second-step and action values derived from the asymmetric Bayesian inference model predicting dopamine activity (GCaMP) in VTA (left), NAc (centre) and DMS (right). Traces reflect mean regression coefficients across subjects; shaded area indicates cross-subject standard error. Dots indicate effect size of the statistically significant timepoints, two-sided t-test comparing the cross-subject distribution against 0, after Benjamini-Hochberg multiple comparison correction.

**Extended Data Fig. 7:**
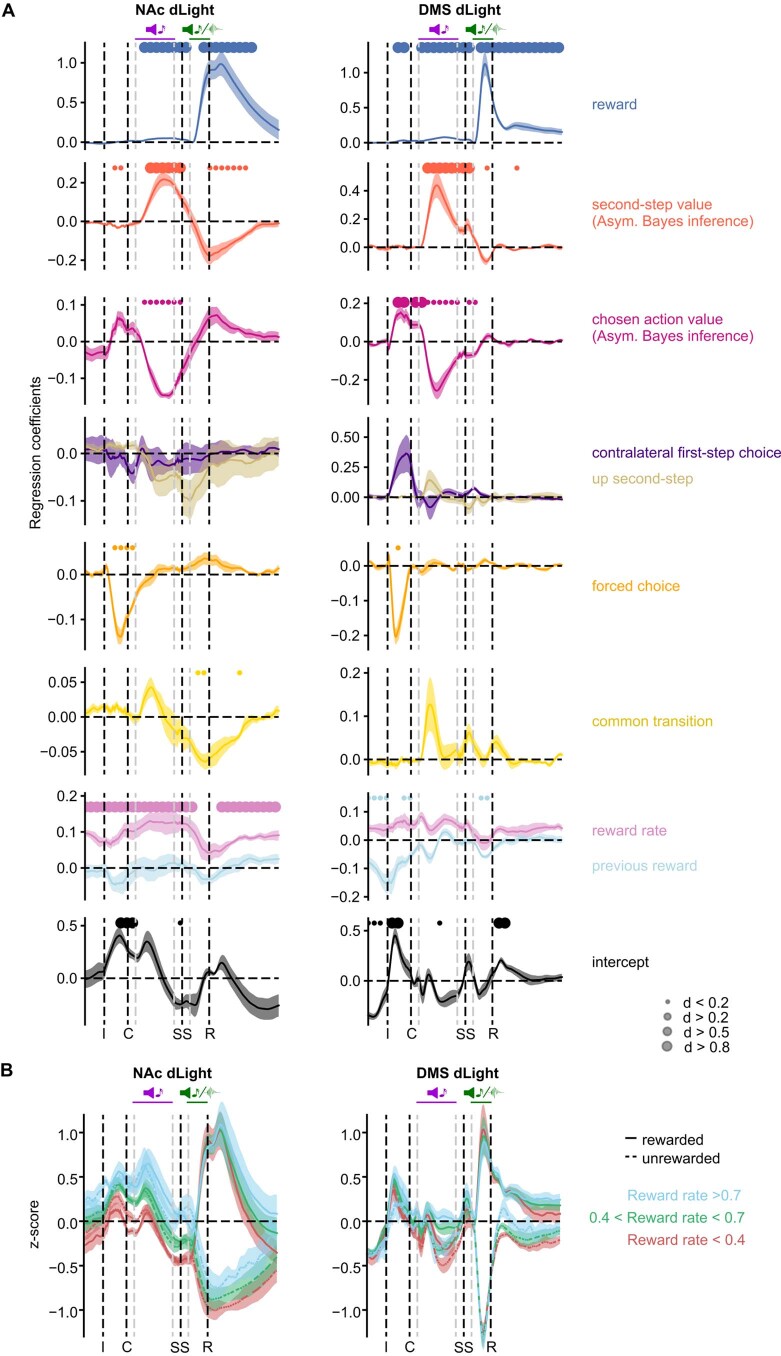
Regression model and reward rate effect in dopamine concentrations. **a)** Regression model using second-step and action values derived from the asymmetric Bayesian inference model predicting dopamine concentrations (dLight) in NAc (left) and DMS (right). Traces reflect mean regression coefficients across subjects; shaded area indicates cross-subject standard error. Dots indicate effect size of the statistically significant timepoints, two-sided t-test comparing the cross-subject distribution against 0, after Benjamini-Hochberg multiple comparison correction. **b)** Mean z-score activity split by rewarded/unrewarded trials and recent reward rate. Shaded area indicates cross-subject standard error. Blue – high reward rate, > 0.7 rewards/trial (exponential moving average with tau = 8 trials). Green, medium reward rate (between 0.4 and 0.7). Red, low reward rate (< 0.4).

Recent work has argued that dopamine fluctuations more closely reflect value than RPE^29^. To examine whether this was the case in our data, we used the same linear regression framework but with value estimates from the inference model for the chosen first-step action and second-step state as predictors (Fig. 4 and Extended Data Figs. 6 and 7). As lateralized movements^6^ and average reward rate^5,29^ have also been reported to influence dopamine, we additionally included regressors for these variables.

In line with our above results, second-step state values drove a biphasic response in NAc GCaMP and dLight signals, with a positive influence when the second-step state was revealed followed by a negative influence at outcome, consistent with RPE not direct value coding (Fig. 4b). VTA GCaMP also showed this biphasic pattern but with a smaller negative response at outcome time relative to the positive response to the second-step state. The time course was more complex in the DMS, with peaks following both the second-step cue and second-step port entry, although the former only survived multiple-comparison correction across timepoints in the dLight data (Extended Data Fig. 7). The chosen action value also had a strong positive influence on activity in all three regions around the time of the choice, which then reversed in all the regions when the second-step value was revealed, again consistent with RPE (Fig. 4c).

In addition to these RPE-like signals, dopamine was also transiently modulated by lateralized movement (Fig. 4d and Extended Data Figs. 4, 6 and 7). Consistent with previous reports^6^, activity in the DMS but not the VTA or NAc was modulated after initiating a contralateral choice. Unlike previous studies, here the task necessitated a second lateralized movement (in the opposite direction) from the choice port back to the centrally located second-step reward port. This did not evoke a response in DMS activity, but did in VTA and NAc activity (note the negative predictor loadings for the VTA and NaC in Fig. 4d following the choice indicate increased activity for contralateral movements from the choice port back to the second-step port).

Reward rate had a strong positive influence on dopamine in all three regions (Fig. 4e and Extended Data Figs. 4, 6 and 7). Unlike the influence of action/state values and rewards, which were tightly time-locked to trial events (Fig. 4a–c), reward rate positively influenced activity at all timepoints, with little modulation by specific trial events (Fig. 4e,f). This reward rate signal was also present in NAc dopamine concentrations, but negligible in DMS concentrations (Extended Data Fig. 7).

In sum, these data demonstrate that dopamine carries information about (i) action and state values in a manner consistent with RPE signaling, (ii) lateralized movement and (iii) recent reward rate. While these signals exist in parallel, they can nonetheless be dissociated based on their timescale and their lateralization.

### Dopamine does not mediate the reinforcing effect of task rewards

**Fig. 5:**
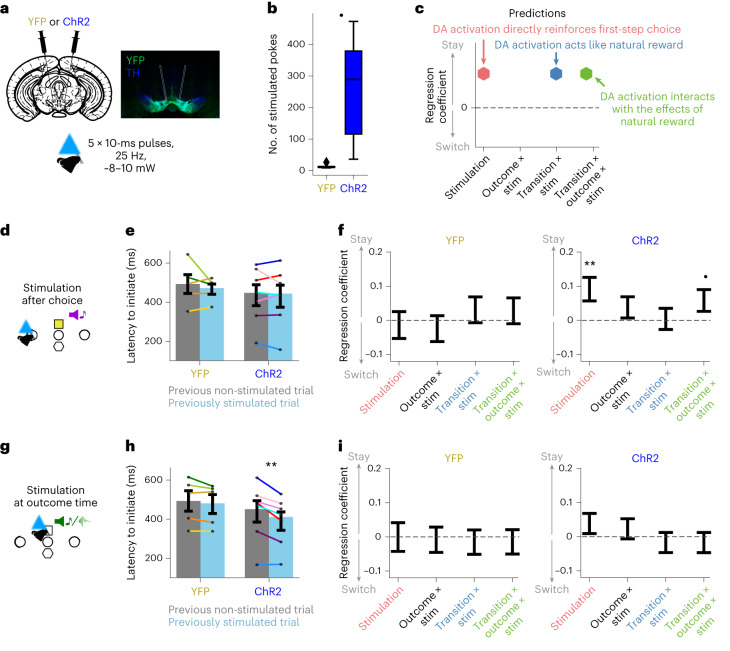
Dopamine stimulation does not recapitulate natural reward effects. **a**, Schematic (left) and photomicrograph (right) showing injection and fiber placement. Photomicrograph of an example ChR2 mouse, stained for tyrosine hydroxylase (TH) and yellow fluorescent protein (YFP). Stimulation consisted of five pulses at 25 Hz, ∼8–10 mW power. **b**, Intracranial self-stimulation. Mean number of entries to a nose-poke port delivering stimulation in the YFP versus the ChR2 group. Error bars show the cross-subject standard error. Box plot shows the distribution of cross-subject regression estimates; box represents the interquartile range, with horizontal lines representing first quartile, median and third quartile, from bottom to top. Whiskers represent minimum and maximum values. Rhomboids mark outliers. *P = 0.011 two-sided independent samples *t*-test. **c**, Predictions on the effect of VTA dopamine (DA) optogenetic activation on animals’ subsequent choices. **d**–**f**, Effect of optogenetic stimulation 200 ms after choice in the control (YFP) and ChR2 groups. **g**–**i**, Effect of optogenetic stimulation at time of the trial outcome cue. **d**,**g**, Optogenetic stimulation schematic. **e**,**h**, Mean latency to initiate a new trial after the center poke illuminates following stimulated and non-stimulated trials. Dots indicate individual animals, and error bars show the s.e. **P = 0.0055, two-sided paired *t*-test. **f**,**i**, Mixed-effects logistic regression predicting repeating the same choice on the next trial using the regression model from Fig. 1e, with additional regressors modeling the effect of stimulation and its interaction with trial events. Only regressors showing stimulation effects are shown here. See Extended Data Fig. 8 for the full model including both groups (YFP and ChR2) and the three stimulation types (non-stim, stim at choice, stim at outcome). Error bars mixed-effects model estimate ± s.d.; statistical significance was assessed using a likelihood-ratio test with type 3 sums of squares. ^•^*P* = 0.069, **P = 0.008. YFP: *n* = 5 animals, 13,079 trials (choice-time stimulation sessions), 15,419 trials (outcome-time stimulation sessions); ChR2: *n* = 7 animals, 20,817 trials (choice-time stimulation sessions), 23,051 trials (outcome-time stimulation sessions).

**Extended Data Fig. 8:**
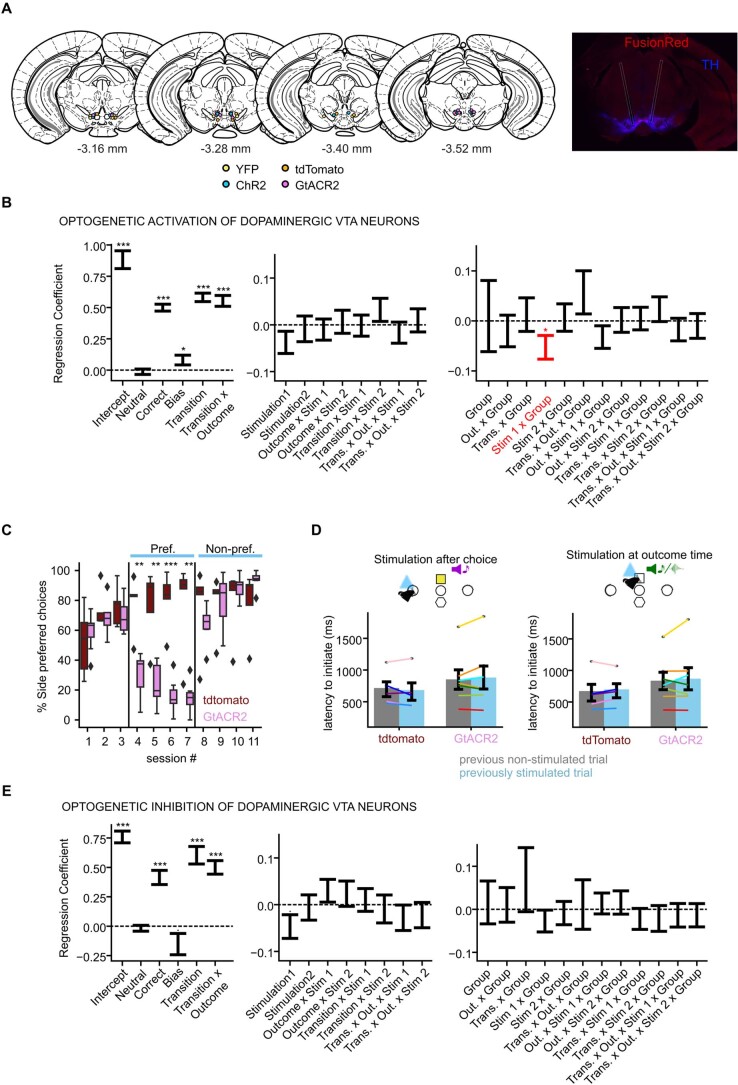
Optogenetic manipulation. **a)** Left, fibre placement. Yellow, YFP animals from the optogenetic activation experiment; blue, ChR2 animals; orange, tdTomato animals from the optogenetic inhibition experiment; pink, GtACR2 animals. Right, photomicrograph showing injection and fibre placement. Photomicrograph comes from an example GtACR2 mouse, stained for TH (Tyrosine Hydroxylase, blue) and FusionRed (red fluorescent protein, red). **b)** Mixed-effects logistic regression predicting stay/switch behaviour in the optogenetic activation experiment. Analysis included data from both groups (YFP and ChR2) and three stimulation types (non-stimulated, stimulation after first-step choice, and stimulation at outcome). Error bars mixed effect model estimate ± s.d.; statistical significance, likelihood-ratio test with Type 3 sums of squares. For exact p-values, see Supplementary Table 6. **c–e)** Optogenetic inhibition experiment. **C)** 2-alternative forced choice control task showing percentage of choices to the initially preferred side in the tdTomato (red) and GtACR2 (pink) groups. Following baseline sessions without stimulation (sessions 1-3), optical stimulation (1s continuous, 5mW) was delivered from sessions 4 to 7 when mice poked their initial preferred choice (preferred side in sessions 1-3). From sessions 8 to 11 the side of optical stimulation was reversed, now to be coincident with choice of their initial non-preferred side. Boxplot show the distribution of cross-subjects regression estimates; box represents the interquartile range, with horizontal lines representing first quartile, median and third quartile, from bottom to top. Whiskers represent minimum and maximum values. Rhomboids mark outliers. **P<0.01, ***P<0.005, two-sided t-test with Bonferroni multiple comparison correction. For exact p-values, see Supplementary Table 6. **D, E)** Inhibition effects on the two-step task. **D)** Mean latency to initiate a new trial after the centre poke illuminates following stimulated and non-stimulated trials. Dots indicate individual animals, error bars show standard error. **E)** As in **B**, but for the optogenetic inhibition experiment, mixed-effects logistic regression including both groups (tdTomato and GtACR2) and three stimulation types (non-stimulated, stimulation after first-step choice, and stimulation at outcome). N: tdTomato: 5 animals, 12,555 trials (choice-time stimulation sessions); 13,373 trials (outcome-time stimulation sessions); GtACR2: 7 animals, 14,789 trials (choice-time stimulation sessions), 15,491 trials (outcome-time stimulation sessions). For exact p-values, see Supplementary Table 6.

To assess the causal influence of dopamine on choices, we manipulated dopamine activity in a new cohort of mice expressing either channelrhodopsin (ChR2; *N* = 7) or a control fluorophore (EYFP, *N* = 5; Fig. 5a and Extended Data Fig. 8a) in VTA dopamine neurons. We verified that our stimulation parameters (five pulses, 25 Hz, ∼8–10 mW power) were sufficient to promote and maintain intracranial self-stimulation in the ChR2 group compared to YFP controls (*t*(10) = 3.107, *P* = 0.011, 95% confidence interval (CI; 68.18, 414.07), Cohen’s *d* = 1.819) using an assay where optical stimulation was delivered contingent on nose-poking in a different context from the two-step task (Fig. 5b).

We then examined the effect on two-step task behavior of optogenetic stimulation at two different timepoints: (i) after the first-step choice, at the time of second-step cue onset, or (ii) at outcome-cue presentation (Fig. 5d,g; 25% stimulated trials, stimulation timepoint fixed for each session, counterbalanced across sessions and mice; *N* = 6–8 sessions per mouse and stimulation type). We again used a mixed-effects logistic regression approach (Fig. 1e), here adding stimulation and its interaction with transition and outcome as regressors. Note that if the effects of rewards are mediated by dopamine, we would expect stimulation to act like a reward, causing positive loading on the transition × stimulation regressor, or, if stimulation interacts with outcome (reward/omission), on the transition × outcome × stimulation regressor; if, however, optogenetic activation simply reinforces the previous choice, this would be evident as positive loading on the stimulation regressor (Fig. 5c).

Data from both groups (YFP and ChR2) and each stimulation type (non-stimulated trials, stimulation after first-step choice and stimulation at outcome) were included in a mixed-effects logistic regression. This revealed a significant stimulation type 1-by-group effect (*β* = −0.053, s.e. = 0.024, *z* = −2230, *P* = 0.026; Extended Data Fig. 8b). To explore this effect, we performed separate logistic mixed-effects regressions for each group and each stimulation type (stimulation after first-step choice and stimulation at outcome).

In the ChR2 group, stimulating dopamine neurons after the first-step choice was reinforcing; it significantly increased the probability of repeating that choice on the next trial (*β* = 0.091, s.e. = 0.035, *z* = 2.645, *P* = 0.008, mixed-effects logistic regression; Fig. 5f). This stimulation did not significantly interact with either the transition or the outcome in its effect on next trial choice (all *P* > 0.069). This is in line with the intracranial self-stimulation result (Fig. 5b) and previous reports^5,7,8,30^ showing that dopamine activation promotes repeating recent actions.

Strikingly, stimulating dopamine neurons at the time of trial outcome—where we observed large increases or decreases in dopamine following reward or omission, respectively—had no significant influence on the subsequent choice (Fig. 5i); it did not reinforce the preceding first-step choice (effect of stimulation: *β* = 0.041, s.e. = 0.030, *z* = 1.378, *P* = 0.168), nor act like a reward by interacting with the state transition (*β* = −0.011, s.e. = 0.030, *z* = −0.355, *P* = 0.723), nor modify the effect outcomes on choices (stimulation–transition–outcome interaction: *β* = −0.014, s.e. = 0.030, *z* = −0.464, *P* = 0.643). No effect of stimulation was found in the YFP group for either stimulation types (all *P* > 0.456). To evaluate the strength of this null result in the ChR2 group, we computed a Bayes factor (*B* = 0.048) for whether dopamine stimulation acted like a task reward or had no effect. This indicated the manipulation result provides ‘strong evidence’ (using the classification in ref. ^31^) against dopamine stimulation recapitulating the behavioral consequences of rewards in this task.

By contrast, while stimulation after the first-step choice had no effect on the latency to initiate the next trial (Fig. 5e, *t*(6) = 0.347, *P* = 0.740, 95% CI (−30.25, 40.25), Cohen’s *d* = 0.034), stimulation at outcome significantly reduced the latency to initiate the next trial (Fig. 5h, *t*(6) = 4.228, *P* = 0.0055, 95% CI (20.98, 78.6), Cohen’s *d* = 0.369). Again, there was no effect on this latency in the YFP group (Fig. 5e, stimulation after choice: *t*(4) = 0.816, *P* = 0.460, 95% CI (−62.7, 114.9), Cohen’s *d* = 0.302; Fig. 5h, stimulation at outcome time: *t*(4) = 1.713, *P* = 0.162, 95% CI (−9.87, 41.67), Cohen’s *d* = 0.141).

To further corroborate that the reward effects observed in the behavior are independent of dopamine, we repeated the previous experiment, but now using either a soma-targeted anion-conducting ChR2 to inhibit VTA dopamine neurons (GtACR2, *N* = 7) or a control fluorophore (tdTomato, *N* = 5; Extended Data Fig. 8a). Our stimulation parameters (1 s continuous, 5 mW) were effective at negatively reinforcing an immediately preceding action in a two-alternative forced-choice control task (Extended Data Fig. 8c). Nonetheless, inhibiting dopamine neurons during the two-step task had no effect on performance (no effect of stimulation by opsin group either in isolation or interacting with other trial events, all *P* > 0.283). To corroborate this null result, we again calculated the Bayes factor for the GtACR2 group at outcome-time stimulation (*B* = 0.062), which indicated ‘strong evidence’ against dopamine inhibition modulating the effects of outcome on subsequent choices.

Therefore, while optogenetic activation or inhibition of VTA dopamine neurons was sufficient to promote or reduce the likelihood of repeating an immediately preceding action, respectively, it completely failed to recapitulate the behavioral consequences of natural rewards at outcome, despite reward delivery and omission driving the largest dopamine signals observed in the task.

### Neural network model reproduces experimental data

Our behavioral and dopamine analyses demonstrate subjects inferred the hidden state of the reward probabilities by observing where rewards were obtained, while our optogenetic manipulations indicate these belief updates (changes in estimates of the hidden state) were not caused by dopamine. This raises several questions: How do subjects learn there is a hidden state that controls the reward probability at both ports? Where and how are beliefs about the hidden state represented and updated? How does state inference interact with the prominent RPE signals we observe in dopamine activity?

**Fig. 6:**
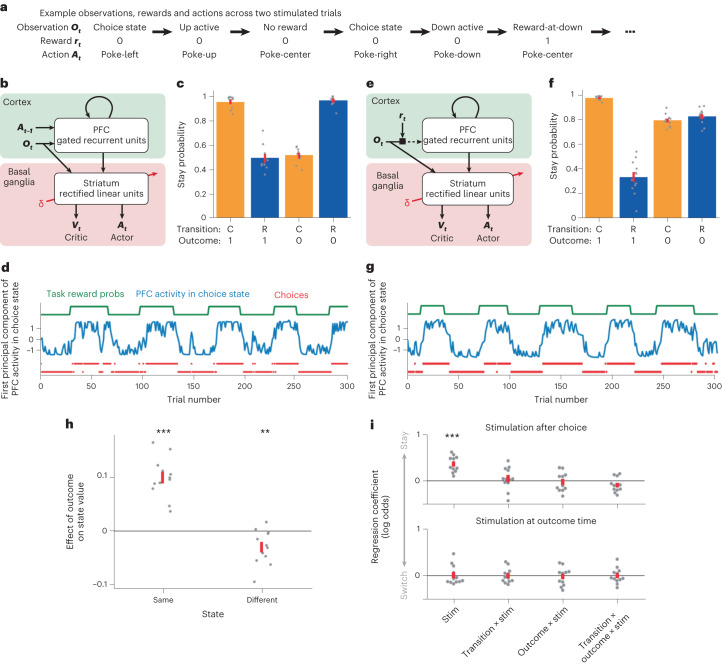
PFC-basal ganglia network model. **a**, Example observations and rewards generated by the task, and actions selected by the model, across two simulated trials comprising six time steps. **b**, Diagram of model used in **c** and **d**. **c**, Stay probability analysis for behavior generated by network model shown in **b**. **d**, Activity of the PFC network in the task’s choice state across trials (blue), projected onto the first principal component of its cross-trial variation. Both PFC activity, and the model choices (red), tracked the state of the task’s reward probability blocks (green). **e**, Diagram of model used in **f** and **g**. The only difference from the model shown in **b** was the input received by the PFC recurrent network. **f**,**g**, As in **c** and **d** but for the model shown in **e**. **h**, Effect of trial outcome (rewarded versus non-rewarded) on the value of the second-step state where reward was received (same) and on the other second-step state (different). ***P = 1.55 × 10^−6^ (same), **P = 0.00798 (different). **i**, Effect of simulated optogenetic stimulation of dopamine neurons, either immediately after taking the first-step choice (top) or at the time of trial outcome (bottom). Stimulation was modeled as modifying weights in the basal ganglia network as by a positive RPE. Extended Data Fig. 9 shows the analyses from **h** and **i** for the model shown in **b**. Individual dots reflect each simulated run (*N* = 12), and error bars indicate the s.e.m. Significance was assessed using a two-sided *t*-test against zero. ***P = 1.33 × 10^−5^.

One possibility is that recurrent networks in cortex learn to infer the hidden states by predicting observations, while RL mechanisms in basal ganglia learn the corresponding value and appropriate action. To test this hypothesis, we implemented a simple neural network model of cortex-basal ganglia circuits (Fig. 6).

The task was modeled as having five actions corresponding to the five nose-poke ports, and five observable states corresponding to trial events (for example, choice state or up-active), such that completing each trial required a sequence of three actions (Fig. 6a). PFC was modeled as a recurrent neural network that received at each time step an observation ***O***_*t*_ (the observable state) and the preceding action ***A***_*t*-1_. PFC activity and observations provided input to a feedforward network representing basal ganglia, comprising a single layer of rectified linear units with two output layers: a scalar estimate of the current value *V*_*t*_ (that is, expected discounted long-run reward) and a vector of action probabilities that determined the next action ***A***_*t*_. The PFC network was trained using gradient descent to predict the next observation given the history of observations and actions. The basal ganglia network was trained using actor-critic RL to estimate the value and select actions given its current input. Network weights were updated gradually across training, and held constant for the simulations analyzed in Fig. 6, such that changes in network activity and behavior from trial to trial were mediated only by the changing input and recurrent activity it induced in PFC.

PFC activity tracked the hidden state of the reward probabilities across trials. Notably, this was true in the choice state (Fig. 6d) even though the next observation in this state does not depend on the reward probabilities (as it is either up-active or down-active). However, to accurately predict trial outcomes, the network must carry information provided by previous outcomes forward through time in its recurrent activity, causing it to be present throughout the trial. The model’s choices tracked the high reward probability option (Fig. 6d), demonstrating that the basal ganglia network was able to use the reward probability information present in its PFC input to select appropriate actions.

Stay probability showed a strong interaction between transition and outcome; that is, the model repeated choices following rewarded common transitions and non-rewarded rare transitions. While this is the pattern expected for an agent that infers the hidden state of the reward probabilities and has a fixed mapping from this to the first-step choice^24^ (Fig. 1g), it can also be generated by model-based RL prospectively evaluating actions by predicting the states they will lead to^24,26^. However, that is not what is happening here: the PFC network only predicts the next observation after an action has been selected, and this prediction is used only for updating PFC network weights.

The model did not exhibit the asymmetric learning from reward and omission observed in the mice. We showed in Fig. 1i,j that models which use Bayesian inference to track the reward probabilities exhibit this asymmetry if they treat rewarded outcomes as distinct observations based on where they occur (that is, top/bottom port), but non-rewarded outcomes as the same observation irrespective of where they occur. We therefore asked if this mechanism could generate such asymmetry in the network model. We modified the input provided to the PFC network such that on each time step PFC received the observation gated by whether reward was received, such that on non-rewarded time steps the input was a zero vector (Fig. 6e). PFC activity and choices still tracked the task reward probabilities (Fig. 6g), but now the stay probabilities recapitulated the asymmetry between rewarded and non-rewarded outcomes seen in the mice (Fig. 6f). As with the explicitly Bayesian models (Fig. 3c), the network model reproduced the nonlocal value updates we observed for the dopamine signal (Fig. 6h): that is, reward in one second-step state increased the value of that state (two-sided *t*-test, *t*(11) = 9.28, *P* < 0.001) but also decreased the value of the other second-step state (two-sided *t*-test, *t*(11) = −3.23, *P* = 0.008).

Finally, we asked how optogenetic stimulation of dopamine on individual trials would affect the model’s choice behavior, assuming it acted by updating weights in the basal ganglia network as if it were a positive RPE (Fig. 6i). Stimulation following first-step choice increased the probability of repeating the choice on the next trial (two-sided *t*-test, *t*(11) = 7.41, *P* < 0.001) as observed experimentally. Crucially, stimulation at trial outcome had no effect on next trial choice (two-sided *t*-test, all |*t*(11)| < 0.30, *P* > 0.77), again recapitulating the data. This is because an RPE following an action updates the network weights to increase the probability of selecting the same action in the same state in future. Therefore, stimulation following a choice affects next trial choice, but stimulation following, for example, an up-poke in the up-active state has no effect on choosing left versus right in the next trial’s choice state.

In sum, the network model recapitulates our key experimental findings that both behavior and the value information that drives dopaminergic RPEs are consistent with state inference, but dopamine does not mediate the effect of rewards on subsequent choices.

## Discussion

By recording and manipulating dopamine activity in a two-step decision task, we obtained results that support an integrated framework for understanding reward-guided decision-making. Rewards did not simply reinforce preceding choices, but rather promoted choosing the action that commonly led to the state where the reward was obtained, consistent with previous work with similar tasks^25,32^. Dopamine carried rich information about value, action and recent reward history, responding strongly and transiently to both rewards and states that predicted reward. The influence of state values on mesolimbic dopamine exhibited a key signature of an RPE^27,28,33^: a positive response when the state was entered followed by a negative response at trial outcome. Additionally, rewards obtained in one second-step state negatively influenced the dopamine response upon reaching the other second-step state on the subsequent trial, consistent with an inferred value update. Strikingly, however, neither optical activation nor inhibition of dopamine neurons at the time of trial outcome—when dopamine responses were maximal—influenced next trial choice, despite positive controls in the same subjects verifying the manipulations were effective.

These findings are not consistent with value updates driven by dopaminergic RPEs changing subject’s choice preference from trial to trial. The observed nonlocal value updates are consistent with the animals understanding that a single hidden variable controls both reward probabilities. However, if animals solve the task by state inference, how do they learn to track the hidden state, and what function do the observed dopaminergic RPEs serve^34^?

Our computational model suggests a possible answer. A recurrent network representing frontal cortex learned to track the hidden state of the reward probabilities by predicting observations. A feedforward network representing basal ganglia learned values and appropriate actions (‘policies’) over the observable and inferred state features using RL, generating choices that closely resembled those of the subjects. Crucially, the short-timescale effect of rewards on subsequent choices was driven by changes in recurrent activity in the PFC, not synaptic weight changes in either network. Consistent with this, two recent studies found that medial frontal and retrosplenial cortex activity tracks the reward probabilities during probabilistic reversal learning, not only at decision or outcome time, but also throughout the intertrial interval^35,36^. This is necessary if recurrent activity is responsible for carrying forward information about the recent reward history to guide choices. This simple model also reproduced our key findings of nonlocal value updates, and the sensitivity of choices to optogenetic stimulation at different timepoints.

This two-process account of reward-guided decision-making can help reconcile the burgeoning evidence for state inference regulating both behavior^10,12–16^ and neural activity in cortex^13,37–42^ and the dopamine system^14,19–21,43,44^, with the long-standing literature supporting dopamine activity resembling and acting like an RPE signal^1–4^. It also helps explain previous findings that although stimulating/inhibiting dopamine following an action can bidirectionally modulate the probability of repeating the action in future^5–8^, inhibiting dopamine at outcome time can fail to block the effect of rewards on subsequent choices^5,6^, and pharmacological manipulation of dopamine signaling in reward-guided decision tasks often has limited or no effect on learning^10,45–47^.

Our network model is related to a recent proposal that frontal cortex acts as a meta-RL system^48^. In both models, synaptic plasticity acts on a slow timescale over task acquisition to sculpt recurrent network dynamics that generate adaptive behavior on a fast timescale. Unlike this previous work, our model differentiates between cortex and basal ganglia, both with respect to network architecture (recurrent versus feedforward) and type of learning (unsupervised versus reinforcement). This builds on long-standing ideas that the cortex implements a hierarchical predictive model of its sensory inputs^49,50^, while the basal ganglia implement temporal-difference RL^2,51^. It is also motivated by work in machine learning in which Markovian state representations (which integrate the observation history to track hidden states) are learned by predicting observations^52–55^, enabling RL to solve tasks where the current observation is insufficient to determine the correct action. There are also commonalities with connectionist models of learning phenomena where one stimulus changes the meaning of another, including occasion setting, configural and contextual learning^56,57^. These use hidden units between an input and output to allow modulatory interactions between stimuli^58,59^, just as in our model the hidden units in basal ganglia allow for nonlinear combination of the current observation and PFC activity to determine value and action.

Recent work has questioned the relative influence of value and RPE on dopamine activity and striatal concentrations^4,5,29^. We observed the biphasic influences of the values of first-step actions and second-step states, a key signature of RPE, in VTA, NAc and DMS calcium activity (Fig. 3 and Extended Data Figs. 4 and 6), and NAc and DMS dopamine concentrations (Extended Data Figs. 4 and 7). This biphasic pattern was most prominent in the NAc. Intriguingly, when evaluating only the influence of the most recent outcome on second-step state value, rather than the extended history, the biphasic pattern was prominent in the NAc but absent in the VTA (Fig. 3e). Differences between the VTA and striatal signals could reflect local modulation of terminals, for example, by cholinergic neurons^60^, or alternatively the VTA signal may be influenced by calcium activity in dendrites that is partially decoupled from spiking due to somatic inhibition.

In parallel, we observed two other important modulators associated with dopamine function—recent reward rate and movement—both of which accounted for separate variance from the value of trial events. Reward rate had positive sustained effects across the trial from before initiation to after outcome, unlike the influence of state and action values which were tightly time-locked to the corresponding behavioral event. This appears broadly consistent with theoretical proposals that tonic dopamine represents average reward rate, which acts as the opportunity cost of time in average reward RL^61^. Reward rate signaling may mediate the effect of dopamine manipulations on motivation and task engagement observed in other studies^46^. The correlation between reward rate and NAc dopamine concentrations is consistent with recent reports^5,29^, but that with VTA calcium is unexpected given previous reports of no correlation with VTA spikes^29^.

By contrast, the influence of movement was transient, lateralized and exhibited distinct dynamics in the DMS and the VTA/NAc. Specifically, DMS, but not NAc or VTA, dopamine was selectively influenced at the time of the initial choice, with increased activity in the hemisphere contralateral to the movement direction consistent with previous studies^6^. Intriguingly, at the time of the second movement from the lateralized choice port to the reward port, significant modulations were instead observed in the NAc (with a similar pattern in the VTA), but not in the DMS, again with increased dopamine activity contralateral to the movement direction. This suggests that an interplay of dopamine dynamics across striatum might shape movement direction as animals proceed through a sequence to reward.

## Conclusion

Our findings emphasize that flexible behavior involves two processes operating in parallel over different timescales: inference about the current state of the world, and evaluation of those states and actions taken in them. The involvement of dopamine in updating values has rightly been a major focus of accounts of flexible decision-making. However, in the structured environments common both in the laboratory and the real world, it is only half the picture. Our data show that during reward-guided decision-making by experienced subjects, the effect of rewards on choices is due to the information they provide about the state of the world, not the dopaminergic RPEs they generate.

## Methods

### Subjects

All procedures were performed in line with the UK Animal (Scientific Procedure) Act 1986 and in accordance with the University of Oxford animal use guidelines. They were approved by the local ethical review panel at the Department of Experimental Psychology, University of Oxford, and performed under UK Home Office Project Licence P6F11BC25. Twelve DAT-Cre heterozygous mice (DAT-Cre^+/−^, 7 females and 5 males) were used for the GCaMP photometry recordings, 6 wild-type C57BL/6 mice (DAT-Cre^−/−^, 3 females and 3 males) for the dLight recordings, and 12 DAT-Cre mice (DAT-Cre^+/−^, YFP: 2 females and 3 males; ChR2: 4 females and 3 males) for the optogenetic activation experiment, and 12 DAT-Cre mice (DAT-Cre^+/−^, tdTomato: 3 females and 2 males; GtACR2: 4 females and 3 males) for the optogenetic inhibition experiment. All animals were bred by crossing DAT-Cre male with C57BL/6 female mice (Charles River, UK). Mice were aged 8–16 weeks at the start of behavioral training. Animals were typically housed in groups of 2–4 throughout training and testing. Temperature was kept at 21 ± 2 °C under 55% ± 10% humidity on a 12-h light–dark cycle. Animals were tested during the light phase.

### Behavioral setup

The task was run in custom-built 12 × 12 cm operant boxes (design files at https://github.com/pyControl/hardware/tree/master/Behaviour_box_small/) controlled using pyControl^62^. Five nose-poke ports were located on the back wall of the boxes—a central initiation port flanked by two choice ports 4 cm to the left and right and two second-step ports located 1.6 cm above and below the central poke. The second-step ports each had a solenoid to deliver water rewards. A speaker located above the ports was used to deliver auditory stimuli. Video data were acquired from an FLIR Chameleon 3 camera positioned above each setup using a Bonsai based workflow (https://github.com/ThomasAkam/Point_Grey_Bonsai_multi_camera_acquisition/)^63^.

### Behavioral task and training

The behavioral task was adapted from the human two-step task^26^. Each trial started with the central initiation port lighting up. Subjects initiated the trial by poking the illuminated port, which caused the choice ports to illuminate. On free-choice trials (75% of trials), both the left and right port lit up, allowing subjects to choose, while on forced-choice trials only one randomly selected choice port lit up, forcing animals to select that specific port. Poking a choice port was followed, after a 200-ms delay, by the second-step port lighting up and 1-s presentation of one of two auditory cues (‘second-step cue’, either a 5-kHz or a 12-kHz tone depending on whether the top or bottom second-step port became active, counterbalanced across animals). Each choice port was commonly (80% trials) associated with transitioning to one second-step state (up or down) and rarely (20% trials) to the other. The transition structure was fixed for each animal across all sessions, and was counterbalanced across animals, that is, the task had two possible transition structures: transition type A, where a left choice commonly led to the up second-step port, and a right choice commonly led to the down second-step port; and the opposite for transition type B. The second-step port only became responsive to pokes after cue offset. Poking the second-step port triggered a 200-ms delay, after which a 500-ms auditory cue signaled whether the trial was rewarded or not (same 5-kHz or 12-kHz tone as the second-step cue, counterbalanced across animals, with pulses delivered at 10 Hz on rewarded trials, white noise on unrewarded trials). Reward was delivered at the offset of this cue. To ensure mice knew when they had made a nose poke, a click sound was presented whenever the subject poked a port that was active (for example, the initiation port during the initiation state).

Reward probabilities for the two second-step ports changed in blocks. In non-neutral blocks, one second-step port had 80% reward probability and the other had 20% probability, while in neutral blocks both second-step ports were rewarded with 50% probability. Block transitions from non-neutral blocks were triggered 5 to 15 trials after mice crossed a threshold of 75% ‘correct’ choices (that is, choosing the higher reward probability), computed as the exponential moving average with a time constant of 8 free-choice trials. Transitions from neutral blocks were triggered after 20–30 trials. An intertrial interval of 2–4 s in duration started once the subject remained out of the second-step port for 250 ms after the trial outcome.

### Training

Behavioral training took 4–6 weeks. Animals were put on water restriction 48 h before starting training, and received 1 h ad-lib water access in their home cage 24 h before starting training. On training days (typically 6 d per week) animals usually received all their water from the task, but were topped up outside the task as necessary to maintain a body weight of >85% of their pre-restriction baseline. On days off from training, mice received 1 h ad-lib water access in their home cage. Water reward size was decreased from 15 µl to 4 µl across training to increase the number of trials performed and blocks experienced on each session.

Training consisted of multiple stages with increasing complexity (Supplementary Table 4). At the first training stage 1.1, only the second-step ports were exposed, with all other ports covered. Second-step ports were illuminated in a pseudorandom order with an intertrial interval of 2–4 s. Poking an illuminated port delivered reward with 100% probability, with no auditory cues. When animals obtained >50 rewards on this stage, they transitioned to stage 1.2, where the auditory cues for second-step state and reward were introduced. Once animals obtained >70 rewards in a session, they were switched to stage 2 on the next session. At stage 2, the choice ports were introduced, but all trials were forced choice, such that only one choice port lit up on each trial. Mice were switched to stage 3 when they obtained >70 rewards on a single session. At stage 3, the initiation poke was introduced, and when they obtained >70 rewards on a single session, they transitioned to stage 4 on the next training session. Stage 4 consisted of multiple substages where progressively more free-choice trials were introduced, and the reward probabilities gradually changed until reaching the final task parameters. Mice were transitioned to the next substage after two training sessions of 45 min or a single 90-min session, until they reached substage 4.6. Subjects were only transitioned to the final stage (full task) when they completed at least 5 blocks in a single session.

### Surgery

Mice were anesthetized with isoflurane (3% induction, 0.5–1% maintenance), and injected with buprenorphine (0.08 mg per kg body weight), meloxicam (5 mg per kg body weight) and glucose saline (0.5 ml). Marcaine (maximum of 2 mg per kg body weight) was injected into the scalp and before placing mice into the stereotaxic frame. Mice were maintained at ∼37 °C using a rectal probe and heating blanket (Harvard Apparatus). Surgery proceeded as described below for the different experiments. After surgery, mice were given additional doses of meloxicam each day for 3 d after surgery, and were monitored carefully for 7 d after surgery.

### GCaMP photometry

Mice were intracranially injected with 1 µl of saline containing a 1:10 dilution of AAV1.Syn.Flex.GCaMP6f.WPRE.SV40 (titer of 6.22 × 10^12^ viral genomes per ml (vg/ml), Penn Vector Core) and a 1:20 dilution of AAV1.CAG.Flex.tdTomato.WPRE.bGH (AllenInstitute864; titer of 1.535 × 10^12^ vg/ml, Penn Vector Core) at 2 nl s^−1^ in VTA (anteroposterior (AP): −3.3, mediolateral (ML): ±0.4, dorsoventral (DV): −4.3 from bregma) in one hemisphere for mesolimbic dopamine recordings in the VTA and NAc and VTA/substantia nigra pars compacta (AP: −3.1, ML: ±0.9, DV: −4.2 from bregma) in the other hemisphere for DMS recordings. Three 200-µm-diameter ceramic optical fibers were implanted chronically in each animal in the VTA (AP: −3.3, ML: ±0.4, DV: −4.3 from bregma) and the NAc (AP: +1.4, ML: ±0.8, DV: −4.1 from bregma) in the same hemisphere, and DMS (AP: +0.5, ML: ±1.5–1.7, DV: −2.6 from bregma) in the contralateral hemisphere.

### dLight photometry

Mice were intracranially injected with 500 nl of saline containing a 1:5 dilution of pAAV5-CAG-dLight1.1 (titer of 1.4 × 10^12^ vg/ml; Addgene) and a 1:5 dilution of pssAAV-2/5-hSyn1-chI-tdTomato-WPRE-SV40p(A) (titer of 4.9 × 10^11^ vg/ml; ETH Zurich) at 2 nl s^−1^ in the NAc (AP: +1.4, ML: ±0.8, DV: −4.1 from bregma) and DMS (AP: +0.5, ML: ±1.5/1.7, DV: −2.6 from bregma) in opposite hemispheres. Two 200-µm-diameter ceramic optical fibers were implanted chronically in the injection sites.

### Optogenetic manipulation

For optical activation experiments, mice were injected bilaterally with 500 nl per hemisphere of saline containing either AAV2-EF1a-DIO-EYFP (titer of >1 × 10^12^ vg/ml, UNC Vector Core; YFP group) or rAAV2/Ef1a-DIO-hchR2(E123t/T159C)-EYFP (titer of 5.2 × 10^12^ vg/ml, UNC Vector Core; ChR2 group) at 2 nl s^−1^ in the VTA (AP: −3.3, ML: ±0.4, DV: −4.3 from bregma). For optical inhibition experiments, mice were injected bilaterally with 500 nl per hemisphere of saline containing a 1:10 dilution of either ssAAV-1/2-CAG-dlox-tdTomato(rev)-dlox-WPRE-bGHp(A) (titer of 7.9 × 10^12^ vg/ml; ETH Zurich; tdTomato group) or AAV1-hSyn1-SIO-stGtACR2-FusionRed (titer of 1.9 × 10^13^ vg/ml; Addgene) (GtACR2 group) at 2 nl s^−1^ in the VTA (AP: −3.3, ML: ±0.4, DV: −4.3 from bregma). For both sets of experiments, two 200-µm-diameter ceramic optical fibers were implanted chronically targeting the injection sites at a 10° angle.

### Histology

Mice were terminally anesthetized with sodium pentobarbital and transcardially perfused with saline and then 4% paraformaldehyde solution. Then, 50-µm coronal brain slices were cut, covering striatum and VTA, and immunostained with anti-GFP and anti-TH primary antibodies, and Alexa Fluor 488 and Cy5 secondary antibodies. For the animals used on the optogenetic inhibition experiment, only anti-TH and Cy5 primary and secondary antibodies, respectively, were used. An Olympus FV3000 microscope was used to image the slices.

### Photometry recordings

Dopamine calcium activity (GCaMP) and release (dLight) were recorded at a sampling rate of 130 Hz using pyPhotometry^64^. The optical system comprised a 465-nm and a 560-nm LED, a five-port minicube and fiber-optic rotary joint (Doric Lenses) and two Newport 2151 photoreceivers. Time division illumination with background subtraction was used to prevent cross-talk between fluorophores due to the overlap of their emission spectra, and changes in ambient light from affecting the signal. Synchronization pulses from pyControl onto a digital input of the pyPhotometry board were used to synchronize the photometry signal with behavior^64^.

**Extended Data Fig. 10:**
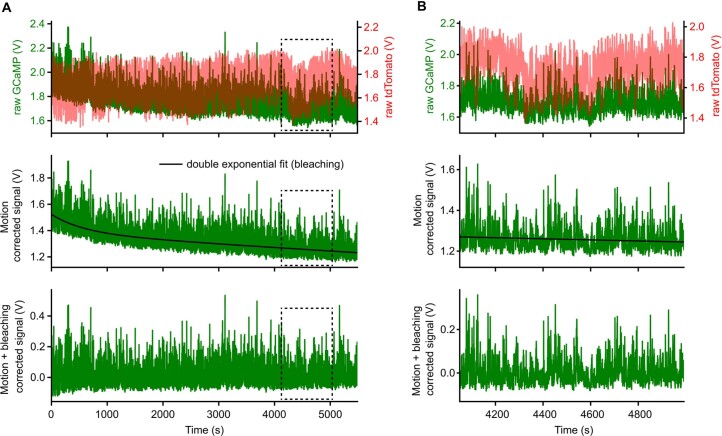
Photometry signal pre-processing. **a, b)** Pre-processing steps in an example session. **A)** Whole recording session. **B)** Zoomed signal from the dashed area in **A**. Top, raw GCaMP and tdTomato signal. Middle, Signal after motion correction, and black line reflects the double exponential fit that will be used for the bleaching correction. Bottom, Motion and bleaching corrected signal.

Photometry signals were pre-processed using custom Python code. A median filter (width of five samples) was first used to remove any spikes due to electrical noise picked up by the photodetectors. Afterwards, a 5-Hz low-pass filter was used to denoise the signal. To obtain motion correction of the signals, we band-passed the denoised signals between 0.001 Hz and 5 Hz, and used linear regression to predict the GCaMP or dLight signal using the control fluorophore (tdTomato) signal. The predicted signal due to motion was subtracted from the denoised signal. To correct for bleaching of the fiber and fluorophores, detrending of signals was performed using a double exponential fit to capture the temporal dynamics of bleaching: a first fast decay and a second slower one (Extended Data Fig. 10). Finally, the pre-processed dopamine signal was *z*-scored for each session to allow comparison across sessions and animals with different signal intensities.

For GCaMP, we recorded data from 12, 11 and 12 mice in VTA, NAc and DMS, respectively (in one animal, the fiber targeting the NAc did not exhibit any GCaMP modulation, which later histological analysis confirmed was caused by the fiber being in the anterior commissure). In the DMS, two animals—the two with most medial coordinates—were excluded from the main analyses of the effects of reward on subsequent dopamine activity as they presented a negative modulation to reward (Extended Data Fig. 3).

For dLight, we recorded data from 5 and 6 mice in the NAc and DMS, respectively (one mouse in the NAc did not present any dLight modulation; subsequent histological analysis confirmed that the fiber was misplaced into the ventricle).

Sessions in which there were large artifacts (large step change in recorded signals) introduced through a malfunctioning of the rotary joint or disconnection of the patch cord from the fiber, or where there was a complete loss of signal on one of the channels due to discharged battery during recording, were excluded. A total of 46 sessions (∼9% of the total) were removed from the analyses.

### Optogenetic activation

VTA dopamine neurons were stimulated bilaterally using two 465-nm LEDs (Plexon Plexbright) connected to 200-µm 0.66-NA optical fiber patch cords. All stimulation experiments used stimulation parameters of five pulses at 25 Hz, 25% duty cycle and ∼8–10 mW optical power at the fiber tip.

First, as a positive control, we performed an intracranial self-stimulation assay in which mice were presented with either 4 or 2 nose-poke ports, one of which triggered optical stimulation when poked. A minimum 1-s delay was imposed between stimulations. Mice were tested on intracranial self-stimulation during 40–60-min sessions for 4 d.

We then tested the effect of optogenetic activation during the two-step task. On each stimulation session, we either stimulated (i) 200 ms after the first-step choice at the time of second-step cue onset, or (ii) at outcome-cue onset. Stimulation occurred on 25% of the trials, under the constraints that (i) the trial after stimulation was always a free-choice trial, and (ii) there were always at least two non-stimulated trials after each stimulation. The stimulation sessions were interspersed with baseline no-stimulation sessions (data not shown). The timing of stimulation was fixed within a session, with the session order counterbalanced across animals (for example, no-stimulation session → second-step cue stimulation session → outcome stimulation session → no-stimulation session → outcome stimulation session → second-step cue stimulation session).

### Optogenetic inhibition

VTA dopamine neurons were inhibited bilaterally using two 465-nm LEDs (Plexon Plexbright) connected to 200-µm 0.66-NA optical fiber patch cords. All inhibition experiments used stimulation parameters of 1-s continuous light at ∼5 mW optical power at the fiber tip.

We first tested the effect of optogenetic inhibition on the two-step task. As in the stimulation experiment, on each inhibition session, we either inhibited (i) 200 ms after the first-step choice at the time of second-step cue onset, or (ii) at outcome-cue onset; baseline sessions with no stimulation were interspersed with the stimulation sessions (data not shown). Inhibition occurred on 25% of the trials under the same constraints as in the optogenetic activation experiment (see above).

As a positive control, we then performed a two-alternative forced-choice bias assay. Mice were presented with three ports: a central initiation port, and left and right choice ports. Mice initiated each trial by poking the illuminated center port. This triggered either both the left and right ports to light up (free-choice trials, 50% of total) or just one choice port to illuminate. Poking an illuminated choice port led, after a 200-ms delay, to 500-ms presentation of an outcome cue (5-kHz or 12-kHz tone—left or right frequency tone counterbalanced across animals—pulsed with 10 Hz on rewarded trials, white noise on unrewarded trials), after which, on rewarded trials, reward was delivered. The reward probability associated with choice of either the left or right port was fixed at 50% throughout. After 3 d of training without optical stimulation and once animals showed a consistent bias toward one of the choice ports for at least two consecutive days (termed the animal’s preferred choice), stimulation sessions commenced. In these, 1 s of continuous light stimulation was delivered coincident with the outcome cue on any trial when the preferred choice port was selected (on both free-choice and forced-choice trials). After 4 d, the light stimulation was then switched to be paired with selection of the other choice port for four more days. Each day, animals were tested on a single 60-min session.

### Analysis

All behavioral and photometry analysis was performed using custom Python and R code.

#### Behavioral logistic regression model

The logistic regression analysis shown in Fig. 1e predicted repeating choices (or ‘staying’) as a function of the subsequent trial events, considering only free-choice trials, implemented as a mixed-effects model using the afex package^65^ in R programming language. The model formula was:

$$\mathrm{{stay}={correct}+{bias}+{transition}\times {outcome}+\left({random}\,{effects}|{subject}\right)}$$

For the analysis of two-step optogenetic manipulation (Fig. 5f,i and Extended Data Fig. 8), we added stimulation (stim) and group and its interaction with trials events as additional predictors, giving the formula:

$$\begin{array}{l}{\mathrm{stay}}={\mathrm{correct}}+{\mathrm{bias}}+{\mathrm{transition}}\times{\mathrm{outcome}}\times{\mathrm{stim}}\times{\mathrm{group}}\\+({\mathrm{random}}\; {\mathrm{effects}}||{\mathrm{subject})}\end{array}$$

We used orthogonal sum-to-zero contrasts, and likelihood-ratio test to calculate *P* values. The maximal random effect structure^66,67^ with subject as a grouping factor was used.

The predictors were coded as:

- Correct: Three-level categorical variable indicating whether the previous choice was correct, incorrect or in a neutral block.
- Bias: Binary categorical variable explaining whether the previous choice was left or right.
- Outcome: Binary categorical variable indicating whether the previous trial was rewarded or not.
- Transition: Binary categorical variable indicating whether the previous trial had a common or a rare transition.
- Stimulation: Three-level categorical variable indicating whether the previous trial was non-stimulated, stimulated after choice or stimulated at outcome time.
- Group*:* Binary categorical variable indicating whether the data are from the control or manipulation subject. YFP and ChR2 were used for the stimulation experiment, and tdTomato and GtACR2 were used for the inhibition experiment.

For the optogenetic manipulation analysis, we performed a single regression for the activation experiment including both experimental groups (YFP and ChR2) and both stimulation times (after choice and outcome). We did the same for the inhibition experiment (tdTomato and GtACR2). To ensure stimulated and non-stimulated trials had matching histories, we only included trials where stimulation could have potentially been delivered, that is, excluding the two trials following each stimulation where stimulation was never delivered. As a follow-up analysis, we performed separate regressions per group (YFP, ChR2, tdTomato and GtACR2) and stimulation time (after choice and outcome).

To test the strength of evidence of our null results, we performed Bayes factor calculation using R as:

$$B=\frac{p({\mathrm{data}}{\rm{|}}{H}_{1})}{p({\mathrm{data}}{\rm{|}}{H}_{0})}$$

We defined the data likelihood as a normal distribution with the mean and standard deviation of the transition × stimulation regression coefficient. For the optogenetic activation experiment, the alternative hypothesis, *H*_1_, was that dopamine activation acted like a natural reward, defined as a uniform distribution between 0 and the transition × outcome regression coefficient. For the optogenetic inhibition experiment, *H*_1_ was that dopamine inhibition reduced the effects of natural rewards, defined as a uniform distribution between 0 and minus the transition × outcome regression coefficient. Finally, the null hypothesis, *H*_0_, was set to 0. We used the classification in ref. ^31^ to assess the strength of evidence for the alternative or null hypothesis.

The lagged logistic regression analysis (Fig. 1f,h) assessed how subjects’ choices were affected by the history of trial events over the last 12 trials. The regression predicted subjects’ probability of choosing left, using the following predictors at lags 1, 2, 3–4, 5–8, 9–12 (where lag 3–4, for example, means the sum of the individual trial predictors over the specified range of lags).

- Common transition: rewarded at lag *n*: +0.5/−0.5 if the *n*th previous trial was a left/right choice followed by a common transition and reward, and 0 otherwise.
- Rare transition: rewarded at lag *n*: +0.5/−0.5 if the *n*th previous trial was a left/right choice followed by a rare transition and reward, and 0 otherwise.
- Common transition: unrewarded at lag *n*: +0.5/−0.5 if the *n*th previous trial was a left/right choice followed by a common transition and no reward, and 0 otherwise.
- Rare transition: unrewarded at lag *n*: + 0.5/−0.5 if the *n*th previous trial was a left/right choice followed by a rare transition and no reward, and 0 otherwise.

The lagged regression was fitted separately for each subject as a fixed-effects model. The cross-subject mean and s.e. for each predictor coefficient were plotted. Significance of coefficients was assessed using a two-sided *t*-test comparing the distribution of the individual subjects’ coefficients against zero, and Bonferroni multiple-comparison correction was performed.

#### Single-strategy models

We evaluated the goodness of fit to subjects’ choices for a set of different RL agents created by combining one or more of the following learning strategies.

##### Model-free

The model-free strategy updated value *Q*_MF_(*c*) of the chosen action and value *V*(*s*) of second-step state as:

$${Q}_{{\mathrm{MF}}}(c){\rm{\leftarrow }}(1-\alpha ){Q}_{{\mathrm{MF}}}(c)+\alpha ((1-\lambda )V(s)+\lambda r)$$

$$V(s){\rm{\leftarrow }}(1-\alpha )V(s)+\alpha r$$

Where *α* is the learning rate, *λ* is the eligibility trace parameter and *r* is the outcome.

A variation of this model was the asymmetric model-free strategy, which included different learning rates for positive and negative outcomes, and included forgetting of the non-experienced states, so both the non-experienced action and second-step state decayed towards neutral value (0.5).

##### Model-based

The model-based strategy updated value *V*(*s*) of the second-step reached, and both first-step action values *Q*_MB_(*a*) as:

$$V(s)\leftarrow(1{\rm{-}}\alpha)V(s)+\alpha r$$

$${Q}_{{\mathrm{MB}}}(a)=\sum _{s}P(s|a)V(s)$$

Where *α* is the learning rate, *r* is the outcome, and *P*(*s*|*a*) is the transition probability of reaching second-step state *s* after taking action *a* (that is, 0.8 or 0.2).

A variation of this model was the asymmetric model-based strategy, which included different learning rates for positive and negative outcomes, and included forgetting of the non-experienced second-step state, which decayed toward a neutral value (0.5). In addition, we tested another model in which the forgetting rate decayed to 0 (asymmetric model-based + forget to 0).

##### Bayesian inference

The Bayesian inference strategy treated the task as having a binary hidden state *h* ∈{up_good, down_good}, which determined the reward probabilities given the second-step state reached on the trial as:

**Table:** 

		Outcome *r*	
*P*(*r*|*s,up_good*)		Rewarded	Unrewarded
Second-step state *s*	Up	0.8	0.2
	Down	0.2	0.8

		Outcome *r*	
*P*(*r*|*s*,down_good)		Rewarded	Unrewarded
Second-step state *s*	Up	0.2	0.8
	Down	0.8	0.2

The strategy maintained an estimate *P*(up_good) tracking the probability the task was in the up_good hidden state, updated following each trial’s outcome using Bayes rule as:

$$P(\mathrm{up}\_\mathrm{good})\leftarrow \frac{P({r|s},\mathrm{up}\_\mathrm{good})P(\mathrm{up}\_\mathrm{good})}{P(r)}$$

Where

$$\begin{array}{l}P(r)=P(r|s,\mathrm{up}{{\_}}\mathrm{good})P(\mathrm{up}{{\_}}\mathrm{good})\\+P(r|s,\mathrm{down}{{\_}}\mathrm{good})P(\mathrm{down}{{\_}}\mathrm{good})\end{array}$$

*P*(up_good) was also updated in each trial based on the probability a reversal occurred as:

$$P(\mathrm{up}\_\mathrm{good})\leftarrow (1-P(\mathrm{reversal}))P(\mathrm{up}\_\mathrm{good})+P(\mathrm{reversal})P(\mathrm{down}\_\mathrm{good})$$

Where *P*(reversal) is the probability a block reversal occurred.

The value of second-step states and first-step actions was determined by *P*(up_good) as:

$$\begin{array}{l}{V}_{\mathrm{inf}}(s)=P(r=1|s,\mathrm{up}{\rm{\_}}\mathrm{good})P(\mathrm{up}{\rm{\_}}\mathrm{good})\\\qquad\qquad+P(r=1|\,s,\mathrm{down}{\rm{\_}}\mathrm{good})P(\mathrm{down}{\rm{\_}}\mathrm{good})\end{array}$$

$${Q}_{\inf }(a)=\sum _{s}P\left(s|a\right){V}_{{inf}}\left(s\right)$$

Note that although the equation relating first-step action values *Q*(*a*) to second-step state values *V*(*s*) is the same for the inference and model-based RL strategies, the mechanistic interpretation is different: For the inference strategy, the action values are assumed to have been learned gradually over task acquisition using temporal difference RL operating over a state representation combining the observable state *s* and belief state *P*(up_good). This learning process was modeled explicitly in the network model (Fig. 6), which generated choice behavior similar to the inference model (Fig. 1). For the model-based RL strategy, the first-step action values are assumed to be computed online by predicting the states the actions will lead to.

We also used a variant of the inference strategy designed to capture the asymmetric influence of reward and omission on subject’s choices. This treated rewards in each second-step state as different observations but treated reward omission at the up and down second-step states as the same observation; implemented as a generative model, which determined the joint probability of reward and second-step state conditioned on the hidden state:

**Table:** 

		Outcome *r*	
*p*(*r*,*s*|up_good)		Rewarded	Unrewarded
Second-step state *s*	Up	0.4	0.5
	Down	0.1	

		Outcome *r*	
*p*(*r,s*|down_good)		Rewarded	Unrewarded
Second-step state *s*	Up	0.1	0.5
	Down	0.4	

The corresponding Bayesian update to *P*(up_good) given each trial’s outcome was given by:

$$P(\mathrm{up}\_\mathrm{good})\leftarrow \frac{P(r,{s|\mathrm{up}{{\_}}{\mathrm{good}}})P(\mathrm{{up}{{\_}}{good}})}{P(r,s)}$$

Where

$$\begin{array}{l}P(r,s)=P(r,s|\mathrm{{up}{\rm{\_}}{good}})P(\mathrm{{up}{\rm{\_}}{good}})\\+P(r,s|\mathrm{{down}{\rm{\_}}{good}})P(\mathrm{{down}{\rm{\_}}{good}})\end{array}$$

State and action values (*V*_inf_ (*s*) and *Q*_inf_ (*a*)) for the asymmetric inference strategy were computed as for the standard inference strategy.

##### Combined action values

A set of different candidate models was created by combining one of more of the above strategies in a weighted sum with (optionally) bias and perseveration parameters, to give net action values:

$${Q}_{\mathrm{{net}}}\left(a\right)=\sum _{i}{w}_{i}{Q}_{i}(a)+K(a)$$

Where *w*_*i*_ is the weight assigned to strategy *i* whose first-step action values are given by *Q*_*i*_ (*a*), and *K*(*a*) is the modifier to the value of first-step action *a* due to any bias or perseveration terms included in the model. In models where bias was included, this increased the value of the left action by an amount determined by a bias strength parameter on all trials. In models where perseveration was included, this increased the value of the first-step action chosen on the previous trial by an amount determined by a perseveration strength parameter.

The combined action values determined choice probabilities via a softmax decision rule as:

$$P\left(a\right)=\frac{{e}^{{Q}_{\mathrm{{net}}}\left(a\right)}}{\sum _{a}{e}^{{Q}_{\mathrm{{net}}}\left(a\right)}}$$

#### Model fitting and comparison

We generated a total of 59 different individual models from the following classes:

Model-free (MF): These models used only the model-free strategy, and varied with respect to whether they had asymmetric learning rates for positive and negative outcomes (together with forgetting toward neutral), and whether they included perseveration or multi-trial perseveration and/or bias.

Model-based (MB): These models used only the model-based strategy, and varied with respect to whether they had asymmetric learning rates for positive and negative outcomes (together with forgetting to either neutral or zero), and whether they included perseveration or multi-trial perseveration and/or bias.

Hybrid (MF + MB): These models used both the model-based and model-free strategies, and varied with respect to whether they had asymmetric learning rates for positive and negative outcomes (together with forgetting toward neutral), and whether they included perseveration or multi-trial perseveration and/or bias.

Bayesian inference: These models used the Bayesian inference strategy, and varied with respect to whether they included an asymmetric updating based on the outcome received, and whether they included perseveration or multi-trial perseveration and/or bias.

Bias increased the value of the left action by an amount determined by a bias strength parameter. Perseveration increased the value of the first-step action chosen on the previous trial by an amount determined by a perseveration strength parameter; in the case of multi-trial perseveration, an exponential moving average of previous choices was used rather than just the previous choice, with a time constant determined by the alpha multi-trial perseveration parameter.

Each model was fit separately to data from each subject using maximum likelihood. The optimization was repeated 30 times starting with randomized initial parameter values drawn from a beta distribution (*α* = 2, *β* = 2) for unit range parameters, gamma distribution (*α* = 2, *β* = 0.4) for positive range parameters and normal distribution (*σ* = 5) for unconstrained parameters, and the best of these fits was used. Model comparison was done using both Bayesian information criterion (Extended Data Fig. 1a and Supplementary Tables 1 and 2) and cross-validated log likelihood using 10 folds (Extended Data Fig. 1b).

To compare data simulated from the single-strategy models with real data (Extended Data Fig. 1g–j), we simulated the same number of sessions for each animal (28.9 ± 1.6 sessions, mean ± s.d.) of their average number of trials for each session (351.6 ± 93.0 trials, mean ± s.d.), using parameters values from each animal’s fits.

#### Mixture-of-strategies model

We created a mixture-of-strategies model, which contained the different behavioral strategies from the single-strategy models (model-free RL, model-based RL and Bayesian inference) as components (Extended Data Fig. 1c–f and Supplementary Table 3). All three components included asymmetric updating from rewards and omissions.

This mixture-of-strategies model combined the action values of the three strategies in a weighted sum with bias and multi-trial perseveration to give net action values as:

$${Q}_{\mathrm{{net}}}(a)=\sum _{i}{w}_{i}{Q}_{i}(a)+K(a)$$

Where *w*_*i*_ is the weight assigned to strategy *i* whose first-step action values are given by *Q*_*i*_(*a*), and *K*(*a*) is the modifier to the value of first-step action *a* due to bias and multi-trial perseveration. The combined action values determined choice probabilities via a softmax decision rule like in the single-strategy models.

This model was fit separately to data from each subject using maximum a posteriori probability, with priors: beta distribution (*α* = 2, *β* = 2) for unit range parameters, gamma distribution (*α* = 2, *β* = 0.4) for positive range parameters and normal distribution (σ = 5) for unconstrained parameters. The optimization was repeated 50 times starting with randomized initial parameter values drawn from the prior distributions.

To test whether behavior generated by the model-based and inference strategies could be differentiated, we fitted the mixture-of-strategies model to data simulated from each single-strategy model using parameters fit to subjects’ data (Extended Data Fig. 1c,d).

#### Photometry analysis

Photometry signals were aligned across trials by linearly time-warping the signal at the two points in the trial where timings were determined by subject behavior; between initiation and choice, and between the second-step port illuminating and being poked (Fig. 2b). Activity at other time periods was not warped.

For the analyses presented in Figs. 3 and 4 and Extended Data Figs. 4–7, we used Lasso linear regression to predict trial-by-trial dopamine activity at each timepoint in a trial as:

$$y(i,t)=\sum _{p}{\beta }_{p}(t){X}_{p}(i)+{\beta }_{o}(t)+\varepsilon(i,t)$$

where *y*(*i, t*) is the calcium *z*-scored activity on trial *i* at timepoint $t$, ${\beta }_{p}(t)$ is the weight for predictor $p$ at timepoint $t$, ${X}_{p}(i)$ is the value of the predictor $p$ on trial *i*, ${\beta }_{o}(t)$ is the intercept at timepoint $t$, and $\varepsilon(i,{t})$ is the residual unexplained variance.

The linear regression was fit separately for each subject to obtain the coefficient time courses *β*_*p*_(*t*). The penalty used for the Lasso regularization was found for each individual regression through cross-validation. When regularization was used, predictors were standardized by centering the mean at 0 and scaling to a variance of 1. For each predictor, we plotted the mean and s.e. across subjects. The statistical significance of each predictor at each timepoint was assessed using a *t*-test comparing the distribution of coefficients across subjects with zero, with Benjamini–Hochberg correction for comparison of multiple timepoints. Effect sizes were computed using Cohen’s *d* at each timepoint as:

$$\mathrm{{Cohe}{n}^{{\prime} }s\,{\it{d}}=\frac{{Mean}\,{regression}\,{coefficient}}{{Standard}\,{deviation}\,{of}\,{regression}\,{coefficient}}}$$

The linear regressions in Fig. 3 and Extended Data Fig. 4 used the following predictors:

- Reward: +0.5 if current trial is rewarded, and −0.5 otherwise.
- Previous reward: +0.5 if previous trial is rewarded, and −0.5 otherwise.
- Good second step: +0.5/−0.5 if the second step reached on the current trial has high/low reward probability, and 0 if neutral block.
- Previous good second step: +0.5/−0.5 if the second step reached on the previous trial has high/low reward probability, and 0 if neutral block.
- Correct choice: +0.5/−0.5 if subject current trial choice commonly leads to the high/low reward probability second-step port, and 0 if neutral block.
- Repeat choice: +0.5 if same choice as previous choice, and −0.5 if different choice to previous trial.
- Direct reinforcement action value update: +0.5/−0.5 if current choice is the same as the previous choice and the previous trial was rewarded/not rewarded, and 0 if different choice from previous trial.
- Inferred action value update: +0.5/−0.5 if current choice commonly leads to the previous second step when it was rewarded/not rewarded, and −0.5/+0.5 if current choice rarely leads to the previous second step when it was rewarded/not rewarded.
- Previous reward, same second step: +0.5/−0.5 if current second step is the same as in the previous trial and previous trial was rewarded/unrewarded, and 0 if current second step is different from the second step on the previous trial.
- Previous reward, different second step: +0.5/−0.5 if current second step is different from the previous second step and previous trial was rewarded/unrewarded, and 0 if current second step is the same as the second step on the previous trial.
- Common transition: +0.5 if a common transition occurs on the current trial, and −0.5 if a rare transition occurs.
- Forced choice: +0.5 if a forced-choice trial occurs on the current trial, and −0.5 if a free-choice trial occurs.
- Reward rate: exponential moving average of the recent reward rate (tau = 10 trials).
- Contralateral choice: +0.5 if the current choice is in the contralateral side from the recording site, and −0.5 if the current choice is in the ipsilateral side.
- Up second step: +0.5 if the current second step is up, and −0.5 if current second step is down.

The linear regression in Extended Data Fig. 5c,d included the same predictors as above (Fig. 3 and Extended Data Fig. 4) except predictors ‘same second step’, ‘previous reward’, ‘different second step’ and ‘inferred action value update’, which were replaced with predictors split by outcome as:

- Same versus different second step, previously rewarded: +0.5/−0.5 if current second step is the same/different as in the previous trial and the previous trial was rewarded, and 0 if the previous trial was not rewarded.
- Same versus different second step, previously non-rewarded: +0.5/−0.5 if current second step is the same/different as in the previous trial and the previous trial was not rewarded, and 0 if the previous trial was rewarded.
- Inferred action value update from rewarded trials: +0.5/−0.5 if current choice commonly/rarely leads to the previous second step and the previous trial was rewarded, and 0 if the previous trial was not rewarded.
- Inferred action value update from unrewarded trials: −0.5/+0.5 if current choice commonly/rarely leads to the previous second step and the previous trial was not rewarded, and 0 if the previous trial was rewarded.

The linear regressions used in Fig. 4 and Extended Data Figs. 6 and 7 used the above-described reward, previous reward, reward rate, contralateral choice, up second step, common transition and forced-choice regressors with the following additional regressors:

- Second-step value: the value ${V}_{{inf}}(s)$ of the second step reached on the current trial from the asymmetric Bayesian inference model.
- Chosen action value: the value ${Q}_{{inf}}(c)$ of the first-step action chosen on the current trial from the asymmetric Bayesian inference model.

Finally, the lagged photometry regression in Extended Data Fig. 5b predicted dopamine response (500 ms at the end of the second-step cue, baseline subtracted using the 500 ms before choice) to the second-step cue as a function of the extended history of trials over the previous 12 trials. No regularization was used in this linear regression. The analysis included the above-described regressors: ‘previous reward, same second step’, ‘previous reward, different second step’, ‘direct reinforcement action value update’ and ‘inferred action value update’ regressors at different lag $n$, with the following additional regressors to correct for correlations in the signal:

- Same versus different second step: +0.5/−0.5 if current second step is the same/different as in the $n$ th previous trial.
- Reward on trial −1 (not lagged): +0.5 if previous trial is rewarded, and −0.5 otherwise.

### Neural network model

For the neural network modeling (Fig. 6), the task was represented as having five observable states—choice state, up-active, down-active, reward-at-up, reward-at-down and no reward—and five actions corresponding to the five ports—poke-left, poke-right, poke-up, poke-down and poke-center. Completing each trial therefore required a sequence of at least three states and actions (for example, choice-state, poke-left → up-active, poke-up → reward-at-up and poke-center), but could take more steps if the agent chose actions that were inactive in the current state (for example, poke-left in the up-active state).

The neural network model consisted of a recurrent network representing PFC and a feedforward network representing basal ganglia, implemented using the Keras Tensorflow API (https://keras.io/). The PFC network was a single fully connected layer of 16 gated recurrent units^68^. In the version of the model shown in Fig. 6b–d, the PFC network received as input on each time step an observation ${{\boldsymbol{O}}}_{t}$ (the observable task state) and the preceding action ${{\boldsymbol{A}}}_{t-1}$, both coded as one-hot vectors. In the version of the model shown in Fig. 6e–i, the PFC network received as input a vector ${{\boldsymbol{O}}}_{t}^{g}$ which was the observation ${{\boldsymbol{O}}}_{t}$ gated by whether reward was received on that time step: On rewarded time steps the input was a one-hot vector indicating the observation (${{\boldsymbol{O}}}_{t}^{g}={{\boldsymbol{O}}}_{t}$), while on non-rewarded time steps the input was a 0 vector (${{\boldsymbol{O}}}_{t}^{g}={\boldsymbol{0}}$).

The basal ganglia network received as input the observation ${{\boldsymbol{O}}}_{t}$ and the activity of the PFC network units. It comprised a layer of ten rectified linear units with two outputs: a scalar-valued linear output for the estimated value ${V}_{t}$ (that is, the expected discounted future reward from the current time step) and a vector-valued softmax output for the policy (that is, the probability of choosing each of the five actions on the next time step).

The model was trained using episodes which terminated after 100 trials or 600 time steps (whichever occurred first), with network weights updated between episodes. For the version of the model used in Fig. 6b–d the PFC network was trained to predict the observation ${{\boldsymbol{O}}}_{t}$ given the preceding observations and actions. For the version of the model used in Fig. 6e–i, the PFC network was trained to predict the reward-gated observation ${{\boldsymbol{O}}}_{t}^{g}$, which it received as input, given this input on preceding time steps. In both cases, PFC network weights were updated using gradient descent with backpropagation through time, with a mean-squared-error cost function, using the Adam optimizer^69^ with learning rate = 0.01. The basal ganglia network was trained using the advantage actor-critic RL algorithm^70^. Hyperparameters for training the basal ganglia network were: learning rate = 0.05, discount factor = 0.9 and entropy loss weight = 0.05.

For each version of the model, we performed 12 simulation runs, each of 500 episodes, using different random seeds. We used these runs as the experimental unit for statistical analysis (that is, the equivalent of subjects in the animal experiments). Data from the last 10 episodes of each run were used for analyses. We excluded any runs that did not obtain reward above chance level in the last 10 episodes, excluding 2 runs of the model version shown in Fig. 6b–d and no runs of the model variant shown in Fig. 6e–i.

To visualize how the activity of PFC units tracked the reward probability blocks, we took the activity of the PFC units in the task’s *‘*choice state*’* on each trial of an episode, yielding an activity matrix of shape (*n_units*, *n_trials*). We used principal component analysis to find the first principal component of the activities’ variation across trials (a vector of weights over units), then projected the activity matrix onto this, giving the time series across trials (Fig. 6d,g).

To evaluate how rewards modified the value of the second-step states (Fig. 6i), we used the model to evaluate both the second-step state that was actually reached on each trial, and the value the other second-step state would have if it had been reached. We then computed how the trial outcome (reward versus omission) changed the value of the second-step state where it was received, and the other second-step state.

To simulate the effects of optogenetic stimulation of dopamine neurons (Fig. 6i), we randomly selected 25% of trials and for each of these trials computed the update to the basal ganglia network weights that would be induced by a positive RPE occurring either following either the choice action (choice-time stimulation) or the second-step action (outcome-time stimulation). We evaluated how these weight updates affected behavior using linear regression to model the probability of repeating the choice on the next trial (stay probability) as a function of the transition, outcome and whether the trial was stimulated or not.

### Statistics and reproducibility

Sample size was determined using power analyses with significance = 0.05 and power = 0.8, using effect sizes based on our own preliminary data.

As described in ‘Photometry recordings’ and ‘Neural network model’, data were excluded as follows: (i) no data were recorded from the NAc in two animals as they did not present any GCaMP or dLight modulation, and subsequent histology confirmed the fiber targeting the NAc was misplaced in these two mice (over the anterior commissure); (ii) sessions with large artifacts or signal loss due to technical issues during recording sessions (total of 46 sessions, representing ∼9% of the total); and (iii) during simulations using the neural network model, runs that did not obtain reward above chance level in the last ten episodes were excluded (total of two runs in the model from Fig. 6b–d).

The presented GCaMP data were obtained using two different cohorts run at different times. dLight data were obtained from a cohort of mice run alongside the second GCaMP cohort. Optogenetic activation and inhibition experiments were also obtained at different times.

Auditory cues and transition probability structure were randomized and counterbalanced across animals and sexes. For the optogenetic assays, group allocation was also randomized. In both activation and inhibition optogenetics, stimulation sessions were interspersed with baseline no-stimulation sessions. The order of stimulation sessions (whether stimulation happened at choice or outcome time) was counterbalanced across animals.

Data collection and analysis were not performed blind to the conditions of the experiments, but the behavioral apparatus and optogenetic stimulation were fully automated, minimizing experimenter influence.

For the statistical reporting, data distribution was assumed to be normal, but this was not formally tested. Where possible, individual data points are shown.

### Reporting summary

Further information on research design is available in the Nature Portfolio Reporting Summary linked to this article.

## Online content

Any methods, additional references, Nature Portfolio reporting summaries, source data, extended data, supplementary information, acknowledgements, peer review information; details of author contributions and competing interests; and statements of data and code availability are available at 10.1038/s41593-023-01542-x.

### Supplementary information

Supplementary Information Supplementary Tables 1–4.

Reporting Summary

Supplementary Table 5 Exact *P* values for statistical tests in Fig. 1.

Supplementary Table 6 Exact *P* values for statistical tests in Extended Data Figs. 1, 5 and 8.

## Figures and Tables

**Extended Data Fig. 9:**
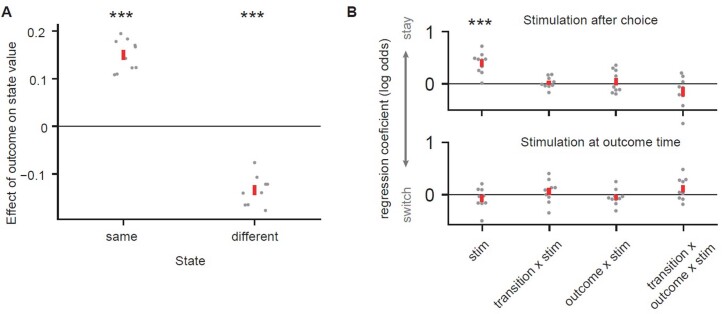
Value updates and simulated dopamine stimulation for version of PFC-basal ganglia network model shown in Fig. 6b. **a)** Effect of trial outcome (rewarded vs non-rewarded) on the value of the second-step state where reward was received (same) and on the other state (different). ***P = 1.41e-7 (same), P=2.00e-7 (different), respectively. **b)** Effect of stimulated optogenetic stimulation after choice (top panel), or at outcome time (bottom panel). Stimulation was modelled as modifying weights in the basal ganglia network as by a positive RPE. N: 10 simulation runs. Two-sided t-test against 0. ***P = 1.36e-4.

## Data Availability

All raw data and pre-processed variables from the manuscript are available on OSF at https://osf.io/u6xrc/.

## Code availability

Complete code used to implement the pyControl task, pre-process the data and generate the Figs. 1–5 is available at https://github.com/Mblancopozo/two-step_dopamine (10.5281/zenodo.10093116).

The code used to simulate the model and generate Fig. 6 is available at https://github.com/ThomasAkam/PFC-BG_model (10.5281/zenodo.10079814).

## References

[1] Montague PR, Dayan P, Sejnowski TJ (1996). A framework for mesencephalic dopamine systems based on predictive Hebbian learning. J. Neurosci..

[2] Schultz W, Dayan P, Montague PR (1997). A neural substrate of prediction and reward. Science.

[3] Eshel N (2015). Arithmetic and local circuitry underlying dopamine prediction errors. Nature.

[4] Kim HR (2020). A unified framework for dopamine signals across timescales. Cell.

[5] Hamid AA (2016). Mesolimbic dopamine signals the value of work. Nat. Neurosci..

[6] Parker NF (2016). Reward and choice encoding in terminals of midbrain dopamine neurons depends on striatal target. Nat. Neurosci..

[7] Steinberg EE (2013). A causal link between prediction errors, dopamine neurons and learning. Nat. Neurosci..

[8] Ilango A (2014). Similar roles of substantia nigra and ventral tegmental dopamine neurons in reward and aversion. J. Neurosci..

[9] Wilson RC, Takahashi YK, Schoenbaum G, Niv Y (2014). Orbitofrontal cortex as a cognitive map of task space. Neuron.

[10] Costa VD, Tran VL, Turchi J, Averbeck BB (2015). Reversal learning and dopamine: a bayesian perspective. J. Neurosci..

[11] Bartolo R, Averbeck BB (2021). Inference as a fundamental process in behavior. Curr. Opin. Behav. Sci..

[12] Vertechi P (2020). Inference-based decisions in a hidden state foraging task: differential contributions of prefrontal cortical areas. Neuron.

[13] Hampton AN, Bossaerts P, O’Doherty JP (2006). The role of the ventromedial prefrontal cortex in abstract state-based inference during decision making in humans. J. Neurosci..

[14] Wimmer GE, Daw ND, Shohamy D (2012). Generalization of value in reinforcement learning by humans. Eur. J. Neurosci..

[15] Baram AB, Muller TH, Nili H, Garvert MM, Behrens TEJ (2021). Entorhinal and ventromedial prefrontal cortices abstract and generalize the structure of reinforcement learning problems. Neuron.

[16] Samborska V, Butler JL, Walton ME, Behrens TEJ, Akam T (2022). Complementary task representations in hippocampus and prefrontal cortex for generalizing the structure of problems. Nat. Neurosci..

[17] Gallistel CR, Mark TA, King AP, Latham PE (2001). The rat approximates an ideal detector of changes in rates of reward: implications for the law of effect. J. Exp. Psychol. Anim. Behav. Process..

[18] Gershman SJ, Niv Y (2010). Learning latent structure: carving nature at its joints. Curr. Opin. Neurobiol..

[19] Bromberg-Martin ES, Matsumoto M, Hong S, Hikosaka O (2010). A pallidus–habenula–dopamine pathway signals inferred stimulus values. J. Neurophysiol..

[20] Babayan BM, Uchida N, Gershman SJ (2018). Belief state representation in the dopamine system. Nat. Commun..

[21] Starkweather CK, Babayan BM, Uchida N, Gershman SJ (2017). Dopamine reward prediction errors reflect hidden-state inference across time. Nat. Neurosci..

[22] Nakahara H, Itoh H, Kawagoe R, Takikawa Y, Hikosaka O (2004). Dopamine neurons can represent context-dependent prediction error. Neuron.

[23] Lak A (2020). Dopaminergic and prefrontal basis of learning from sensory confidence and reward value. Neuron.

[24] Akam T, Costa R, Dayan P (2015). Simple plans or sophisticated habits? State, transition and learning interactions in the two-step task. PLoS Comput. Biol..

[25] Akam T (2021). The anterior cingulate cortex predicts future states to mediate model-based action selection. Neuron.

[26] Daw ND, Gershman SJ, Seymour B, Dayan P, Dolan RJ (2011). Model-based influences on humans’ choices and striatal prediction errors. Neuron.

[27] Behrens TEJ, Hunt LT, Woolrich MW, Rushworth MFS (2008). Associative learning of social value. Nature.

[28] Niv Y, Edlund JA, Dayan P, O’Doherty JP (2012). Neural prediction errors reveal a risk-sensitive reinforcement-learning process in the human brain. J. Neurosci..

[29] Mohebi A (2019). Dissociable dopamine dynamics for learning and motivation. Nature.

[30] Pan WX, Coddington LT, Dudman JT (2021). Dissociable contributions of phasic dopamine activity to reward and prediction. Cell Rep..

[31] Jeffreys, H. *Theory of Probability* (Clarendon Press, 1961).

[32] Miller KJ, Botvinick MM, Brody CD (2017). Dorsal hippocampus contributes to model-based planning. Nat. Neurosci..

[33] Rutledge RB, Dean M, Caplin A, Glimcher PW (2010). Testing the reward prediction error hypothesis with an axiomatic model. J. Neurosci..

[34] Akam T, Walton ME (2021). What is dopamine doing in model-based reinforcement learning?. Curr. Opin. Behav. Sci..

[35] Bari BA (2019). Stable representations of decision variables for flexible behavior. Neuron.

[36] Hattori R, Komiyama T (2022). Context-dependent persistency as a coding mechanism for robust and widely distributed value coding. Neuron.

[37] Schuck NW, Cai MB, Wilson RC, Niv Y (2016). Human orbitofrontal cortex represents a cognitive map of state space. Neuron.

[38] Klein-Flügge MC, Wittmann MK, Shpektor A, Jensen DEA, Rushworth MFS (2019). Multiple associative structures created by reinforcement and incidental statistical learning mechanisms. Nat. Commun..

[39] Bradfield LA, Dezfouli A, van Holstein M, Chieng B, Balleine BW (2015). Medial orbitofrontal cortex mediates outcome retrieval in partially observable task situations. Neuron.

[40] Starkweather CK, Gershman SJ, Uchida N (2018). The medial prefrontal cortex shapes dopamine reward prediction errors under state uncertainty. Neuron.

[41] Bartolo R, Averbeck BB (2020). Prefrontal cortex predicts state switches during reversal learning. Neuron.

[42] Jones JL (2012). Orbitofrontal cortex supports behavior and learning using inferred but not cached values. Science.

[43] Gershman SJ, Uchida N (2019). Believing in dopamine. Nat. Rev. Neurosci..

[44] Sadacca BF, Jones JL, Schoenbaum G (2016). Midbrain dopamine neurons compute inferred and cached value prediction errors in a common framework. Elife.

[45] Grogan JP (2017). Effects of dopamine on reinforcement learning and consolidation in Parkinson’s disease. Elife.

[46] Korn C (2021). Distinct roles for dopamine clearance mechanisms in regulating behavioral flexibility. Mol. Psychiatry.

[47] Eisenegger C (2014). Role of dopamine D2 receptors in human reinforcement learning. Neuropsychopharmacology.

[48] Wang JX (2018). Prefrontal cortex as a meta-reinforcement learning system. Nat. Neurosci..

[49] Rao RP, Ballard DH (1999). Predictive coding in the visual cortex: a functional interpretation of some extra-classical receptive-field effects. Nat. Neurosci..

[50] Friston K (2005). A theory of cortical responses. Philos. Trans. R. Soc. Lond. B. Biol. Sci..

[51] Doya K (2000). Complementary roles of basal ganglia and cerebellum in learning and motor control. Curr. Opin. Neurobiol..

[52] Sutton, R. S. & Barto, A. G. *Reinforcement Learning: an Introduction* (MIT press, 2018).

[53] Littman, M. & Sutton, R. S. Predictive representations of state. In *Advances in Neural Information Processing Systems* (eds. T. Dietterich et al.) **14** (MIT Press, 2001).

[54] Lin, L. & Mitchell, T. M. Reinforcement learning with hidden states. In *From Animals to Animats 2:**Proceedings of the Second**International Conference on Simulation of Adaptive Behavior* (eds Meyer, J.-A., Roitblat, H. L., Wilson, S. W.) (MIT Press, 1993).

[55] Igl, M., Zintgraf, L. M., Le, T. A., Wood, F. & Whiteson, S. Deep variational reinforcement learning for POMDPs. In *Proceedings of the 35th International Conference on Machine Learning* 2117–2126 (2018).

[56] Pearce JM, Bouton ME (2001). Theories of associative learning in animals. Annu. Rev. Psychol..

[57] Fraser KM, Holland PC (2019). Occasion setting. Behav. Neurosci..

[58] Delamater AR (2012). On the nature of CS and US representations in Pavlovian learning. Learn. Behav..

[59] Schmajuk NA, Lamoureux JA, Holland PC (1998). Occasion setting: a neural network approach. Psychol. Rev..

[60] Threlfell S, Cragg SJ (2011). Dopamine signaling in dorsal versus ventral striatum: the dynamic role of cholinergic interneurons. Front. Syst. Neurosci..

[61] Niv Y, Daw ND, Joel D, Dayan P (2007). Tonic dopamine: opportunity costs and the control of response vigor. Psychopharmacology.

[62] Akam T (2022). Open-source, Python-based, hardware and software for controlling behavioural neuroscience experiments. Elife.

[63] Lopes G (2015). Bonsai: an event-based framework for processing and controlling data streams. Front. Neuroinform..

[64] Akam T, Walton M (2019). pyPhotometry: open source Python based hardware and software for fiber photometry data acquisition. Sci. Rep..

[65] Singmann, H., Bolker, B., Westfall, J. & Aust, F. afex: analysis of factorial experiments. R package. (2018).

[66] Barr, D. J., Levy, R., Scheepers, C. & Tily, H. J. Random effects structure for confirmatory hypothesis testing: keep it maximal. *J. Mem. Lang*. **68**, 255–278 (2013). 10.1016/j.jml.2012.11.001 PMC3881361 24403724

[67] Matuschek, H., Kliegl, R., Vasishth, S., Baayen, H. & Bates, D. Balancing type I error and power in linear mixed models. *J. Mem. Lang*. **94**, 305–315 (2017).

[68] Cho, K. et al. Learning phrase representations using RNN encoder-decoder for statistical machine translation. In *Proceedings of the 2014 Conference on Empirical Methods in Natural Language Processing* (eds. A. Moschittiet al.) 1724–1734 (ACL, 2014). 10.3115/v1/d14-1179

[69] Kingma, D. P. & Ba, J. Adam: A method for stochastic optimization. In *Proceedings of the**Third International Conference on Learning Representations* (eds. Bengio, Y. & LeCun, Y.) (2015).

[70] Mnih, V. et al. Asynchronous methods for deep reinforcement learning. In *Proceedings of the**International conference on machine learning* 1928–1937 (2016).

