# Supplementary material for: Dopamine-independent effect of rewards on choices through hidden-state inference
Source: Nat Neurosci. 2024 Jan 12;27(2):286–97. doi: 10.1038/s41593-023-01542-x (PMC10849965; doi:10.1038/s41593-023-01542-x)
Supplement: Supplementary file 1 — Supplementary Tables 1–4. [file 41593_2023_1542_MOESM1_ESM.pdf]

nature neuroscience

Article https://doi.org/10.1038/s41593-023-01542-x

Dopamine-independent effect of rewards on choices through hidden-state inference

In the format provided by the authors and unedited

SUPPLEMENTARY TABLES

Supplementary Table 1. Single strategy models.

	Model Category	BIC	Forget	Sym.	Asym.	Bias	Persev.	Multi-Persev.
**0**	Asym. MB + forget to 0	139831.80	■		■	■		■
**1**	Asym. Bayesian inf.	140036.05			■	■		■
**2**	Asym. MB + forget to 0	140086.30	■		■			■
**3**	Asym. Bayesian Inf.	140276.03			■			■
**4**	Asym. MB + forget	141590.81	■		■	■		■
**5**	Asym. hybrid + forget	141805.52	■		■	■		■
**6**	Asym. MB + forget	141867.80	■		■			■
**7**	Asym. hybrid + forget	142073.50	■		■			■
**8**	Asym. MB + forget to 0	143070.87	■		■	■	■	
**9**	Asym. MB + forget to 0	143775.49	■		■		■	
**10**	Asym. hybrid + forget	144008.16	■		■	■	■	
**11**	Asym. Bayesian Inf.	144239.97			■	■	■	
**12**	Asym. MB + forget	144459.12	■		■	■	■	
**13**	Asym. hybrid + forget	144526.49	■		■	■		
**14**	Asym. hybrid + forget	144544.82	■		■		■	
**15**	Asym. MB + forget to 0	144862.99	■		■	■		
**16**	Asym. Bayesian Inf.	144978.67			■		■	
**17**	Asym. hybrid + forget	145051.16	■		■			
**18**	Asym. MB + forget	145149.48	■		■		■	
**19**	Asym. MB + forget to 0	145728.24	■		■			
**20**	Asym. MB + forget	146579.22	■		■	■		
**21**	Asym. Bayesian Inf.	146813.37			■	■		
**22**	Asym. MB + forget	147405.69	■		■			
**23**	Asym. Bayesian Inf.	147801.67			■			
**24**	Bayesian Inf.	147948.45		■		■		■
**25**	Bayesian Inf.	148229.72		■				■
**26**	MB	149671.55		■		■		■
**27**	Hybrid	149682.46		■		■		■
**28**	MB	150022.51		■				■
**29**	Hybrid	150023.82		■				■
**30**	Asym. MF + forget	153158.95	■		■	■		■
**31**	Asym. MF + forget	153253.21	■		■			■
**32**	Asym. MF + forget	153271.91	■		■	■	■	
**33**	Asym. MF + forget	153517.90	■		■		■	
**34**	Hybrid	153970.40		■		■	■	
**35**	MB	154043.05		■		■	■	
**36**	MF	154115.34		■		■		■
**37**	Bayesian Inf.	154202.23		■		■	■	
**38**	MF	154273.02		■				■
**39**	Asym. MF + forget	154621.49	■		■	■		
**40**	Hybrid	154817.62		■			■	
**41**	Asym. MF + forget	154860.06	■		■			
**42**	Bayesian Inf.	154879.49		■			■	
**43**	MB	154909.12		■			■	
**44**	MF	158491.35		■		■	■	
**45**	Control	158903.27		■				■
**46**	Control	159003.42		■		■		■
**47**	MF	159080.31		■			■	
**48**	Hybrid	165694.02		■		■		
**49**	MB	165920.96		■		■		
**50**	Hybrid	167002.03		■				
**51**	MB	167264.02		■				
**52**	Bayesian Inf.	167728.20		■		■		
**53**	Bayesian Inf.	168835.61		■				
**54**	Control	168847.38		■		■	■	
**55**	Control	168878.81		■			■	
**56**	MF	171713.00		■		■		
**57**	MF	172669.69		■				
**58**	Control	188360.39		■		■		

Abbreviations: Sym. = Symmetric; Asym. = Asymmetric; Inf. = Inference; MB = model based; MF = model free; Persev. = Perseveration; Multi-Persev = Multi-Perseveration.

Supplementary Table 2. Best single strategy model of each class.

Rank	Best models of each class	BIC	parameters	kernels
0	**Asymmetric model-based + forget to 0**	139831.8	weight: 3.24 ± 1.12<br>learning rate reward: 0.63 ± 0.14<br>learning rate non-reward: 0.65 ± 0.14<br>forget: 0.71 ± 0.18	multi-persev. weight: 2.95 ± 0.59<br>multi-persev. alpha: 0.21 ± 0.20<br>bias: 0.01 ± 0.14
1	**Asymmetric Bayesian inference**	140036.05	weight: 23.24 ± 21.55<br>prob. reversal: 0.33 ± 0.07	multi-persev. weight: 3.06 ± 0.43<br>multi-persev. alpha: 0.19 ± 0.10<br>bias: 0.01 ± 0.14
4	**Asymmetric model-based + forget**	141590.81	weight: 4.98 ± 1.45<br>learning rate reward: 0.71 ± 0.14<br>learning rate non-reward: 0.16 ± 0.08<br>forget: 0.83 ± 0.13	multi-persev. weight: 2.98 ± 0.61<br>multi-persev. alpha: 0.23 ± 0.19<br>bias: 0.01 ± 0.14
5	**Asymmetric hybrid + forget**	141805.52	weight model-free: 0.37 ± 0.41<br>weight model-based: 4.81 ± 1.41<br>learning rate reward: 0.68 ± 0.14<br>learning rate non-reward: 0.14 ± 0.06<br>eligibility trace: 0.50 ± 0.42<br>forget: 0.82 ± 0.16	multi-persev. weight: 2.92 ± 0.65<br>multi-persev. alpha: 0.20 ± 0.20<br>bias: 0.01 ± 0.14
24	**Bayesian inference**	147948.45	weight: 5.36 ± 1.90<br>prob. reversal: 0.24 ± 0.05	multi-persev. weight: 3.45 ± 0.51<br>multi-persev. alpha: 0.35 ± 0.16<br>bias: 0.01 ± 0.14
26	**Model-based**	149671.55	weight: 1.55 ± 0.49<br>learning rate: 0.69 ± 0.11	multi-persev. weight: 3.13 ± 0.51<br>multi-persev. alpha: 0.37 ± 0.19<br>bias: 0.00 ± 0.16
27	**Hybrid**	149682.46	weight model-free: 0.25 ± 0.13<br>weight model-based: 1.32 ± 0.44<br>learning rate: 0.78 ± 0.11<br>eligibility trace: 0.17 ± 0.22	multi-persev. weight: 3.13 ± 0.51<br>multi-persev. alpha: 0.37 ± 0.19<br>bias: 0.01 ± 0.15
30	**Asymmetric model-free + forget**	153158.95	weight model-free: 3.06 ± 0.94<br>learning rate reward: 0.56 ± 0.19<br>learning rate non-reward: 0.26 ± 0.20<br>eligibility trace: 0.49 ± 0.15<br>forget: 0.40 ± 0.14	multi-persev. weight: 2.37 ± 1.06<br>multi-persev. alpha: 0.49 ± 0.37<br>bias: 0.01 ± 0.11
36	**Model-free**	154115.34	weight: 1.26 ± 0.40<br>learning rate: 0.46 ± 0.08<br>eligibility trace: 0.53 ± 0.08	multi-persev. weight: 3.06 ± 0.46<br>multi-persev. alpha: 0.39 ± 0.18<br>bias: 0.01 ± 0.12
45	**Control**	158903.27		multi-persev. weight: 3.78 ± 0.45<br>multi-persev. alpha: 0.32 ± 0.11

Supplementary Table 3. Mixture-of-strategies models.

Mixture-of-strategies model – forget to neutral (0.5)

Second-step value update	Action value update	kernels
**Independent**	**Model-free**	multi-persv. weight: 2.97 ± 0.66
learning rate reward: 0.47 ± 0.18	learning rate reward: 0.52 ± 0.17	multi-persv. alpha: 0.21 ± 0.18
learning rate non-reward: 0.27 ± 0.25	learning rate non-reward: 0.46 ± 0.24	bias: 0.01 ± 0.15
forget: 0.59 ± 0.26	eligibility trace: 0.43 ± 0.18	
	weight: 0.67 ± 0.47	
	**Model-based**	
	weight: 1.47 ± 1.29	
**Bayesian inference**	**Bayesian inference**	
prob. reversal: 0.36 ± 0.09	weight: 22.47 ± 15.03	

Mixture-of-strategies model – forget to 0

Second-step value update	Action value update	kernels
**Independent**	**Model-free**	multi-persv. weight: 2.85 ± 0.73
learning rate reward: 0.62 ± 0.15	learning rate reward: 0.53 ± 0.20	multi-persv. alpha: 0.17 ± 0.17
learning rate non-reward: 0.64 ± 0.19	learning rate non-reward: 0.55 ± 0.19	bias: 0.01 ± 0.15
forget: 0.70 ± 0.26	eligibility trace: 0.51 ± 0.18	
	weight: 0.51 ± 0.54	
	**Model-based**	
	weight: 2.35 ± 1.18	
**Bayesian inference**	**Bayesian inference**	
prob. reversal: 0.29 ± 0.20	weight: 5.05 ± 8.88	

Supplementary Table 4. Description of training stages and task contingencies

Stage	Start poke	Choice pokes	Reward pokes	Task Structure
1.1			X	Reward probability 100%
1.2				Introduce auditory cues<br>Reward probability 100%
2		X	X	Transition probability 80/20%<br>Reward probability 100%
3	X	X	X	Transition probability 80/20%<br>Reward probability 100%
	x	x	x	Start introducing free choice trials and reward probabilities
Substage	**Free choice trials**	**Non-neutral blocks reward probabilities** (high / low)	**Neutral block reward probability**	**Block change** (independent / dependent on behaviour)
4.1	0%	0.9/0.7	0.8	Independent – 20-30 trials blocks
4.2	25%	0.9/0.5	0.7	Independent – 20-30 trials blocks
4.3	25%	0.9/0.3	0.6	Independent – 20-30 trials blocks
4.4	25%	0.9/0.1	0.5	Independent – 20-30 trials blocks
4.5	50%	0.9/0.1	0.5	Dependent
4.6	75%	0.9/0.1	0.5	Dependent
4.7 – Final	75%	0.8/0.2	0.5	Dependent
