# Supplementary material for: Dopamine-independent effect of rewards on choices through hidden-state inference
Source: Nat Neurosci. 2024 Jan 12;27(2):286–97. doi: 10.1038/s41593-023-01542-x (PMC10849965; doi:10.1038/s41593-023-01542-x)
Supplement: Supplementary file 2 — Reporting Summary [file 41593_2023_1542_MOESM2_ESM.pdf]

nature portfolio

Corresponding author(s): Mark E. Walton

Last updated by author(s): Nov 9, 2023

Reporting Summary

Nature Portfolio wishes to improve the reproducibility of the work that we publish. This form provides structure for consistency and transparency in reporting. For further information on Nature Portfolio policies, see our Editorial Policies and the Editorial Policy Checklist.

Statistics

For all statistical analyses, confirm that the following items are present in the figure legend, table legend, main text, or Methods section.

n/a	Confirmed	
☐	☒	The exact sample size (*n*) for each experimental group/condition, given as a discrete number and unit of measurement
☐	☒	A statement on whether measurements were taken from distinct samples or whether the same sample was measured repeatedly
☐	☒	The statistical test(s) used AND whether they are one- or two-sided<br>*Only common tests should be described solely by name; describe more complex techniques in the Methods section.*
☐	☒	A description of all covariates tested
☐	☒	A description of any assumptions or corrections, such as tests of normality and adjustment for multiple comparisons
☐	☒	A full description of the statistical parameters including central tendency (e.g. means) or other basic estimates (e.g. regression coefficient) AND variation (e.g. standard deviation) or associated estimates of uncertainty (e.g. confidence intervals)
☐	☒	For null hypothesis testing, the test statistic (e.g. *F*, *t*, *r*) with confidence intervals, effect sizes, degrees of freedom and *P* value noted<br>*Give P values as exact values whenever suitable.*
☐	☒	For Bayesian analysis, information on the choice of priors and Markov chain Monte Carlo settings
☐	☒	For hierarchical and complex designs, identification of the appropriate level for tests and full reporting of outcomes
☐	☒	Estimates of effect sizes (e.g. Cohen's *d*, Pearson's *r*), indicating how they were calculated

Our web collection on statistics for biologists contains articles on many of the points above.

Software and code

Policy information about availability of computer code

Data collection	pyControl v1.8.1 (reference 62) was used to control the behavioural task, acquire behavioural data and manage the optogenetic stimulation. Fibre photometry data was acquired using pyPhotometry v0.3.3 (reference 64).
Data analysis	Data analysis was performed using custom written code in Python (v3.10.0) and R (v4.1.2).<br>Python packages: matplotlib=3.5.1; pingouin=0.5.1; seaborn=0.11.2; numpy==1.22.3; numba==0.55.0; pandas==1.4.1; patsy==0.5.2; parse=1.19.0; rpy2=3.4.5; scikit-learn=1.0.1; scipy=1.7.3; statannotations=0.4.4; statsmodels=0.13.0; more-itertools=8.5.0.<br>R packages: afex=0.28-1; bayesplay=0.9.2.<br><br>Code is available at: https://github.com/Mblancopozo/two-step_dopamine (doi: 10.5281/zenodo.10093116) and https://github.com/ThomasAkam/PFC-BG_model (doi: 10.5281/zenodo.10079814)

For manuscripts utilizing custom algorithms or software that are central to the research but not yet described in published literature, software must be made available to editors and reviewers. We strongly encourage code deposition in a community repository (e.g. GitHub). See the Nature Portfolio guidelines for submitting code & software for further information.

Data

Policy information about availability of data

All manuscripts must include a data availability statement. This statement should provide the following information, where applicable:

- Accession codes, unique identifiers, or web links for publicly available datasets
- A description of any restrictions on data availability
- For clinical datasets or third party data, please ensure that the statement adheres to our policy

Data is available at: https://osf.io/u6xrc/

Field-specific reporting

Please select the one below that is the best fit for your research. If you are not sure, read the appropriate sections before making your selection.

☒ Life sciences ☐ Behavioural & social sciences ☐ Ecological, evolutionary & environmental sciences

For a reference copy of the document with all sections, see nature.com/documents/nr-reporting-summary-flat.pdf

Life sciences study design

All studies must disclose on these points even when the disclosure is negative.

Sample size	We used power analyses with significance = 0.05 and power = 0.8, using effect sizes based on our own preliminary data.
Data exclusions	No data was recorded from NAc in 2 animals as they did not presented any GCaMP or dLight modulation. Later histological confirmed these two mice had the fibre targeting NAc misplaced.<br>Sessions in which there were large artifacts (large step change in recorded signals) introduced through a malfunctioning of the rotary joint or disconnection of the patch cord from the fibre, or where there was a complete loss of signal on one of the channels due to discharged battery during recording, were excluded. A total of 46 sessions (~9% of the total) were removed from the analyses.
Replication	Data presented was obtained using two different cohorts at different times. Therefore, the results were replicated internally.
Randomization	Auditory cues and transition probability structure were randomised and counterbalanced across animals and sexes. For the optogenetic assays, group allocation was also randomised. In both activation and inhibition optogenetics, stimulation sessions were interspersed with baseline no stimulation sessions. The stimulation sessions order (whether stimulation happened at choice or outcome time) was counterbalanced across animals.
Blinding	Data collection and analysis were not performed blind to the conditions of the experiments, but the behavioural apparatus and optogenetic stimulation was fully automated minimising experimenter influence.

Reporting for specific materials, systems and methods

We require information from authors about some types of materials, experimental systems and methods used in many studies. Here, indicate whether each material, system or method listed is relevant to your study. If you are not sure if a list item applies to your research, read the appropriate section before selecting a response.

Materials & experimental systems

n/a	Involved in the study	
☐	☒	Antibodies
☒	☐	Eukaryotic cell lines
☒	☐	Palaeontology and archaeology
☐	☒	Animals and other organisms
☒	☐	Human research participants
☒	☐	Clinical data
☒	☐	Dual use research of concern

Methods

n/a	Involved in the study	
☒	☐	ChIP-seq
☒	☐	Flow cytometry
☒	☐	MRI-based neuroimaging

Antibodies

Antibodies used	Primary antibodies: Rabbit Anti-Tyrosine Hydroxylase Antibody (dil. 1:1000, Sigma-Aldrich, CAT AB152) and Chicken Anti-Green Fluorescent Protein Antibody (dil. 1:500, Aves Labs, CAT GFP-1020).<br>Secondary antibodies: Cy5 Donkey Anti-Rabbit (dil. 1:500, Jackson ImmunoResearch, CAT 711-175-152) and Alexa Fluor 488 Donkey Anti Chicken (dil. 1:1000, Jackson ImmunoResearch, CAT 703-545-155).

Validation

Both primary and secondary antibodies were validated by the manufacturer, and has been cited previously (peer-reviewed citations, CAT AB152: 1064; CAT GFP-1020: 747; CAT 711-175-152: 412; CAT 703-545-155: 864). Additionally, all the antibodies were validated internally in the lab.

Animals and other organisms

Policy information about studies involving animals; ARRIVE guidelines recommended for reporting animal research

Laboratory animals

12 dopamine transporter (DAT)-Cre heterozygous mice (7 females and 5 males) were used for the GCaMP photometry recordings, 6 WT C57BL/6 mice (3 females and 3 males) for the dLight recordings, 12 DAT-Cre mice (YFP: 2 females and 3 males; ChR2: 4 females and 3 males) for the optogenetic activation experiment, and 12 DAT-Cre mice (tdTomato: 3 females and 2 males; GtACR2: 4 females and 3 males) for the optogenetic inhibition experiment. Mice were aged 8-16 weeks at the start of behavioural training. Animals were typically housed in groups of 2-4 throughout training and testing. Temperature was kept at 21 ± 2°C under 55 ± 10% humidity on a 12h light/dark cycle. Animals were tested during the light phase.

Wild animals

This study did not involve wild animals.

Field-collected samples

This study did not involve field-collected samples.

Ethics oversight

All procedures were performed in line with the UK Animal (Scientific Procedure) Act 1986 and in accordance with the University of Oxford animal use guidelines. They were approved by the local ethical review panel at the Department of Experimental Psychology, University of Oxford and performed under UK Home Office Project Licence P6F11BC25.

Note that full information on the approval of the study protocol must also be provided in the manuscript.
